# Prevalence and mortality associated with multidrug-resistant infections in adult intensive care units in Argentina (PREV-AR)

**DOI:** 10.1128/aac.01426-24

**Published:** 2025-01-22

**Authors:** Wanda Cornistein, Carina Balasini, Yanina Nuccetelli, Viviana M. Rodriguez, Norma Cudmani, Maria Virginia Roca, Graciela Sadino, Martín Brizuela, Analía Fernández, Soledad González, Damián Águila, Alejandra Macchi, Maria Inés Staneloni, Elisa Estenssoro, Prevar Study Group, Alexis Manzo

**Affiliations:** 1 Hospital Universitario Austral 218809 , Buenos Aires, Argentina; 2 Argentinian Society of Infectious Diseases (SADI), Buenos Aires, Argentina; 3 Argentinian Society for Critical Care (SATI), Buenos Aires, Buenos Aires, Argentina; 4 Hospital Pirovano, Buenos Aires, Argentina; 5 Hospital San Martín de La Plata, Buenos Aires, Argentina; 6 Hospital Tornú 580023 , Buenos Aires, Argentina; 7 Departamento de Control de Infecciones y Gestión de Antimicrobianos, Ministerio de Salud Publica, Tucuman, Argentina; 8 Hospital Zonal Alvear, Comodoro Rivadavia, Argentina; 9 Clinica Universitaria Reina Fabiola, Cordoba, Argentina; 10 Hospital General de Agudos Velez Sarsfield, Buenos Aires, Argentina; 11 Hospital Durand, Buenos Aires, Argentina; 12 Ministerio de Salud de la Provincia de Buenos Aires 150184 , Buenos Aires, Argentina; 13 Hospital Provincial del Centenario 541448 , Rosario, Argentina; 14 Sanatorio Las Lomas 433090 , Buenos Aires, Argentina; 15 Hospital Italiano de Buenos Aires 37533 , Buenos Aires, Argentina; Houston Methodist Hospital and Weill Cornell Medical College, Houston, Texas, USA

**Keywords:** multidrug resistance, prevalence, mortality, carbapenem-resistant Enterobacterales, adult intensive care units, hospital infections, colonization, Argentina

## Abstract

Data from low and middle-income countries (LMICs) on multidrug-resistant microorganisms (MDROs) in intensive care units (ICUs) are scarce. Working in several ICUs in Argentina, we sought to estimate the prevalence and characteristics of MDRO infections and carbapenemase-producing Enterobacterales (CPE) colonization. Mortality associated with MDRO infection was also evaluated. The study was a 24-hour point prevalence study conducted in 164 adult ICUs in Argentina between 24 November and 28 November 2023. The main study outcome was in-ICU mortality and secondary outcomes included the prevalence of MDRO infections, the prevalence of CPE colonization (defined as CPE recovered from a rectal swab), and ICU length of stay (LoS). Mixed effects modeling was used to identify risk factors for in-ICU mortality. Among 1,799 patients, 933 (51.9%) had a reported infection; 599 infections (64.2%) were classified as definite (i.e., with positive cultures) and 334 (35.8%) as probable infection (i.e., negative cultures but signs of infection). Of the 933 patients with infection, 273 (29.2%) had an MDRO recovered with 344 total MDRO cultures recovered. Non-MDRO was recovered from 326 (34.9%) of the 933 patients. Among definite infections, 45.5% (273/599) were due to MDRO with an overall prevalence of MDRO of 15.1% (273 patients with MDRO infections/1,799 patients). CPE colonization, defined as a positive rectal swab taken during the incident hospitalization, occurred in 420/1,696 (24.7%) patients. The most frequent MDRO infection was ventilator-associated pneumonia (100/344; 29.1%). The most common MDRO recovered were carbapenem-resistant *Acinetobacter baumannii* and CPE (98/344, 28.5% each). In-ICU mortality was 27.1% (487/1,799); independent predictors were age (odds ratio [OR] 1.01 [1.00–1.02], *P* = 0.003), MDRO infection (OR 1.65 [1.18–2.43], *P* = 0.012), probable infection (OR 1.41 [0.97–2.04], *P* = 0.073), sepsis-related organ failure assessment (SOFA) score (OR 1.18 [1.13–1.23], *P* = 0.000), and hospital-acquired pneumonia (OR 1.84 [1.12–3.01], *P* = 0.016). Mortality also varied significantly by hospital (*P* < 0.001). LoS was significantly longer in patients with MDRO infections, 30.0 (interquartile range [IQR] 17–35) days vs 16.0 (IQR 8–33) in non-MDRO, *P* < 0.0001. Among 1,799 ICU patients in an LMIC, the prevalence of MDRO infection and CPE colonization was high. The presence of an MDRO infection was associated with increased mortality and prolonged ICU LoS.

**CLINICAL TRIALS:**

This study is registered with Clinicaltrials.gov as NCT06574776.

## INTRODUCTION

Infection by multidrug-resistant microorganisms (MDROs) represents a great clinical challenge, given their association with increased mortality and morbidity (1, 2). Comprehensive data on MDRO infections, including epidemiology and risk factors, as well as the impact on health outcomes, play a pivotal role in increasing awareness about this issue among physicians, patients, and stakeholders, particularly in resource-limited settings (1). In addition, these data can influence the development of health policies focused on the prevention, diagnosis, and treatment of these infections, and facilitate the adequate allocation of resources (1). Critically ill patients are particularly vulnerable to MDRO colonization and infection, thus awareness of the MDRO prevalence in an intensive care unit (ICU) is essential to the successful design and implementation of interventions to combat their spread (3).

In recent decades, there has been a rise of antimicrobial resistance (AMR) worldwide, especially in South America, where most healthcare facilities are located in settings with limited resources (1, 2, 4). Most studies describing AMR in this region have focused on colonization or infection without evaluating the impact on ICU mortality (1, 2, 5, 6). In addition, most reported data predate the coronavirus disease 2019 (COVID-19) pandemic, after which the incidence of MDRO infections rose steeply worldwide (7). Updated estimates of the epidemiology and clinical impact of MDROs post-COVID-19 pandemic are essential.

In a meta-analysis of data from studies in Latin-American and Caribbean countries from 2000 to 2022, the overall, unadjusted case-fatality rate related to MDROs was 45.0% (2). In a 2019 study that modeled the burden of AMR globally, the estimated number of AMR-associated deaths in the Americas, 569,000 annually, was substantially higher than in high-income regions (1). However, these studies were not limited to ICU patients. A Brazilian study on MDRO-associated ICU mortality in the region demonstrated an in-hospital mortality of 29.5% (8).

To better understand the post-pandemic ICU epidemiology of MDRO infection and colonization as well as the associated mortality of MDROs, the Argentine Societies of Infectious Diseases (SADI) and Intensive Care Medicine (SATI) carried out a cross-sectional study. We hypothesized that the prevalence of infections caused by MDRO among ICU patients would be higher than 30%, as described in a prior study of high-prevalence regions (9).

## MATERIALS AND METHODS

### Study design

PREVAR (PREValence of infection by MDRO in ARgentina) was a national, multicenter, point prevalence study in 164 adult ICUs in Argentina. Data at each site were collected over a 24-hour period with all data collection done between 24 November and 28 November 2023.

### Participants

Hospitals were recruited via announcements at national scientific meetings, professional society websites, and emails to society listservs. Hospitals were registered to participate in the study utilizing a secure website, where local investigators recorded hospital level characteristics in an electronic form.

All adult patients, defined as 18 years and older, who were present in the participating ICU during the 24-hour study period (beginning at 8:00 a.m.) were included. Enrolled patients in the point prevalence study were then followed for in-ICU mortality. There were no exclusion criteria.

### Data collection

Patient data were entered in an electronic case report form (CRF) using the Research Electronic Data Capture (REDCap) database. Local researchers received online training on data collection and the CRF. A centralized data center managed the database and monitored data quality, in collaboration with local researchers, to minimize missing data. Patient data were anonymized by assigning a study ID to each patient in order of admission.

The CRF included baseline demographic characteristics of the study participants, including date of hospital and ICU admission, age, gender, comorbid conditions, and admission APACHE II and sepsis-related organ failure assessment (SOFA) scores. SOFA score was also calculated and recorded on the day of enrollment. Data on risk factors for MDRO infection during the prior 6 months were collected, including prior hospital admission and carbapenemase-producing Enterobacterales (CPE) colonization (either a history of colonization or incident positive rectal swab). Colonization data on CPE were included, vs other MDRO, as it is the most common type of colonization monitored in Argentinian ICUs. Antibiotic utilization and type of admission (medical, elective, or emergency surgery) were also collected.

Patient infection status, on the day of the point prevalence survey, was recorded by the treating physician according to the SEPSIS-3 definitions: without infection, with infection but no sepsis, sepsis, and septic shock (10).

In addition, infections were considered as definite (microbiologically confirmed), and probable or possible, according to the International Sepsis Forum (ISF) definitions for pneumonia, bloodstream infections (including infective endocarditis), intravascular catheter-related sepsis, intra-abdominal infections, urosepsis, and surgical wound infections (11).

In the cases where culture results were pending, categorization was reviewed with the main investigators when the culture results were available. Definitions of infection sites were those provided by the ISF (11). Infections were registered as community-acquired, ICU-acquired, hospital non-ICU acquired, or originating in long-term care facilities.

The CRF allowed the collection of up to four concurrent infections per patient if they were present at enrollment. According to culture results, infections were categorized as MDRO or non-MDRO. MDROs included carbapenem-resistant *Acinetobacter baumannii* (CRAB), difficult-to-treat *Pseudomonas aeruginosa*, CPE, organisms with an extended spectrum β-lactamase (ESBL)-producing phenotype, vancomycin-resistant Enterococci, and methicillin-resistant *Staphylococcus aureus* (Table S1). If available, further diagnostic methods were performed to classify genetic mechanisms of resistance including *Klebsiella pneumoniae* carbapenemase-producing Enterobacterales, metallo-beta-lactamases (MBL), oxacillinases (OXA), ESBL-producing organisms, and AMPC beta-lactamases. Non-MDRO organisms of interest included *Streptococcus pneumoniae, Streptococcus pyogenes, S. aureus, Escherichia coli,* coagulase-negative *Staphylococcus*, *Proteus* sp., *Klebsiella-Enterobacter-Serratia-Citrobacter* (KESC) group, *Clostridium difficile*, among others.

Antimicrobial treatment was classified as empiric or targeted, according to the absence or presence of positive cultures, respectively. Treatment was further classified in several ways. First, treatment was classified as adequate or inadequate, according to antimicrobial susceptibility testing results. Second, it was categorized as utilizing novel vs “old” antibiotics according to the European guidelines for the treatment of infections caused by multidrug-resistant Gram-negative bacilli (12). Finally, treatment was classified into Access, Watch, and Reserve antibiotic groups according to the AWaRe WHO classification (12, 13).

At 60 days post-enrollment, information on mortality status (ICU discharge vs death) was requested from local investigators in each participating ICU. At this time, outstanding data on any pending cultures were also collected. Hospitals were classified as general or specialized and as public or private. We recorded the total number of hospital beds, quantity of ICU beds, and the presence of infection prevention and control committees and antimicrobial stewardship programs. Additionally, data on frequency of MDRO surveillance for infections and colonization were collected. Methods used to detect types of bacterial resistance were recorded as phenotypic, molecular, or immunochromatographic.

### Outcomes

The main study outcome was ICU mortality. Secondary outcomes included the prevalence of MDRO infections, the prevalence of CPE colonization (defined as CPE recovered from a rectal swab), and ICU length of stay (LoS). Data were censored at 60 days.

### Statistical analysis

Prevalence was calculated as the number of patients with MDRO infections in the participating ICU divided by the number of patients in the ICU on the day of the study. Independent variables are expressed as absolute numbers and percentages or as medians and interquartile ranges. We evaluated the differences in independent variables among patients with MDRO infections and non-MDRO infections, between survivors and non-survivors, and between patients with and without infections. Differences were analyzed using the *χ*² test or Fisher’s exact test for categorical variables, and the *t*-test or Wilcoxon rank-sum test for continuous variables, as appropriate.

To estimate the association between patient characteristics, ICU organizational factors, and hospital characteristics and MDRO infection and ICU mortality, we used mixed-effects modeling. These models have a hierarchical structure with patients (level 1) nested within hospitals (level 2), which accounts for the potential correlation of outcomes within the same hospital. Hospitals were treated as random effects to account for the potential correlation of outcomes within the same hospital and to capture variability between hospitals through random intercepts. Patient characteristics and ICU organizational factors were treated as fixed effects, as they were the primary predictors of interest.

Only variables with a *P* value <0.20 in the bivariable analysis were included in the final model. The results for fixed effects are reported as odds ratios (ORs) with 95% confidence intervals (CIs). Random effects, or measures of variation, are reported as the variance, standard error (SE), and the median OR. The statistical significance of covariates was determined using the likelihood ratio test.

All reported *P* values are two-sided, with a *P* value <0.05 considered statistically significant.

## RESULTS

### General characteristics

**Fig 1:**
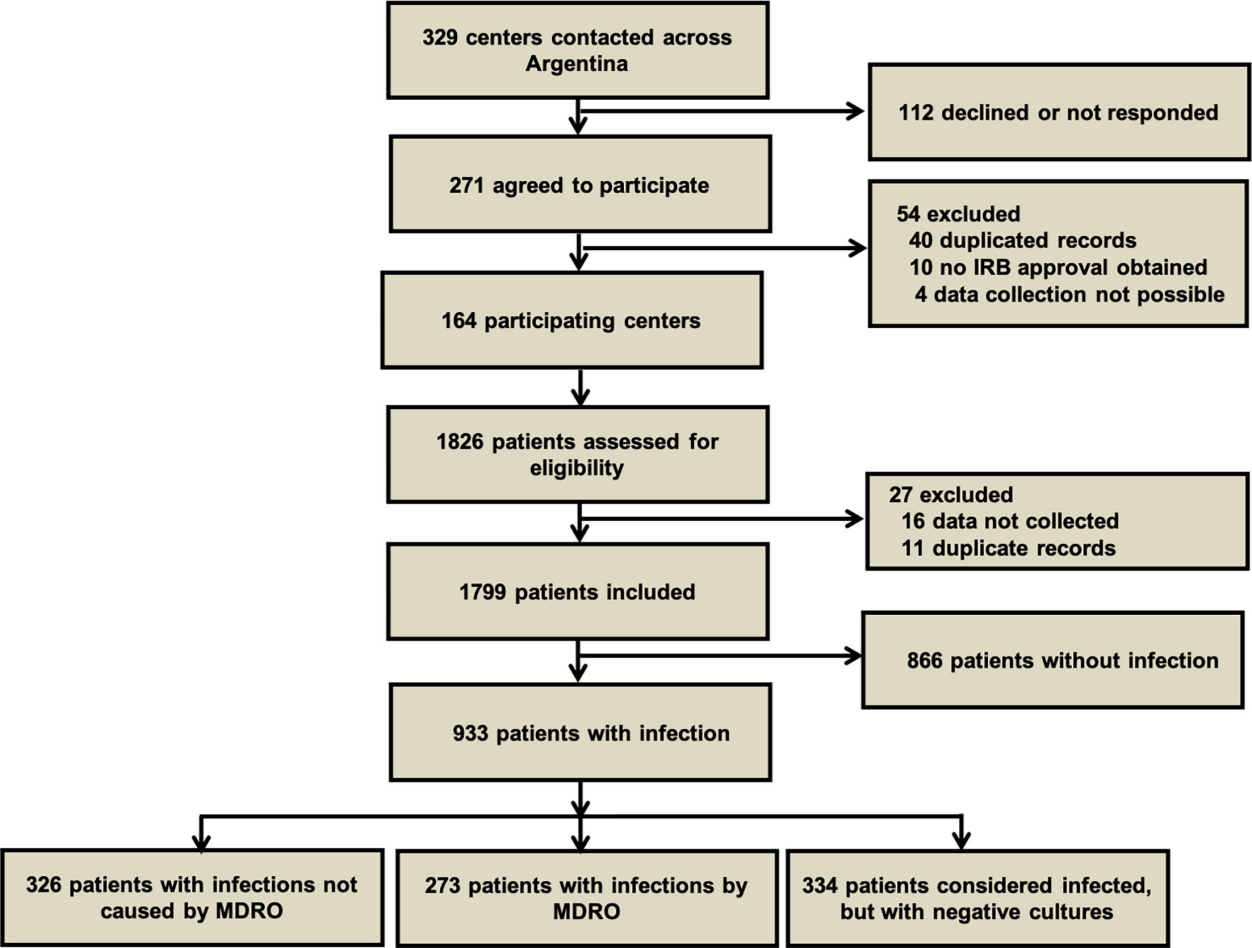
Flow diagram of the study. Each local institutional review board (IRB) defined the requirement for informed consent.

A total of 164 ICUs participated in the study; the recruitment flow diagram is shown in Fig. 1. A majority of hospitals were public (*n* = 104, 63.8%), and almost all were general (*n* = 153, 93.8%). One hundred fifty-four (94.5%) had infection prevention and control committees, and 80 (49.1%) had antimicrobial stewardship programs. Other characteristics are shown in Table 1.

**TABLE 1:** Characteristics of participating hospitals, *N* = 164^*a*^

Characteristic	Value
Hospital category	
Public	104 (63.8%)
Private	49 (30.1%)
Community	1 (0.6)
Health insurance-associated	7 (4.3%)
Other	2 (1.2%)
Type of hospital	
General	153 (93.8%)
Specialized	10 (6.1%)
Number of hospital beds	120 [55–205]
Number of ICU beds	12 [8–18]
Number of patients included per hospital	9 [6–13]
Other resources	
Infection prevention and control committee present	154 (94.5%)
Antimicrobial stewardship program present	80 (49.1%)
Frequency of CPE screening cultures	
Weekly	131 (80.3%)
At ICU admission	81 (49.3%)
Prior to surgery	32 (19.6%)
Methods used to characterize bacterial resistance	
Phenotypic	143 (88.3%)
Molecular	43 (26.3%)
Immunochromatographic	89 (54.6%)

^*a*^ Data are presented as counts (%) or median [0.25–0.75] percentiles.

There were 1,799 patients included for analysis with a median of nine patients [6–13] enrolled per center. Median age was 59 years [43–71], and most patients were male (*n* = 1,118, 62%). The most frequent comorbidities were diabetes (*n* = 412, 22.9%), obesity (*n* = 401, 22.3%), and respiratory disease (*n* = 276, 15.3%). Most patients were classified as medical admissions (*n* = 1,176, 65.3%).

### Prevalence and characteristics of infection

Nine hundred thirty-three patients were categorized as having an infection for a prevalence of 51.9% (933/1,799). 32.8% of infections were determined to be hospital-acquired. Compared with patients without infection, patients with infection had a higher prevalence of obesity, pre-existent respiratory disease, human immunodeficiency virus infection, and alcohol use disorder. Patients with infection also were more likely to have had a hospital admission or CPE colonization in the prior 6 months, and a longer LoS at enrollment (Table S2).

At enrollment, 448/933 (48.0%) infected patients were classified as having infection without sepsis, 308/933 (33.0%) were classified as having sepsis, and 157/933 (16.8%) were classified as having septic shock. Sepsis was categorized as definite in 176/933 patients (18.9%) and probable or possible in 132/933 (14.1%) patients (Table S2). Infection was considered definite in 599 of the 933 patients (64.2%), with 818 positive cultures identified among those 599 patients. Of the positive cultures, 582 (71.1%) were identified as Gram-negative organisms and 236 (28.9%) as Gram-positive.

### Prevalence and characteristics of MDRO infections

Of the 933 enrolled patients, 273 patients had a microbiologically proven MDRO infection (29.3) and 326 had a microbiologically confirmed non-MDRO infection (34.9%). Among the 599 patients with definite infections, 45.5% (273/599) were due to an MDRO, and 54.4% (326/599) were due to a non-MDRO (Table S2).

A subset of the 273 patients with MDRO infections had >1 infection reported at enrollment. Overall, 344 MDRO cultures were recorded among the 273 patients, yielding 1.3 MDRO infections per patient (344/273). The prevalence of MDRO infections in the study population was 15.1% (273 patients with MDRO infections/1,799 patients).

Patients with MDRO infections were younger, more likely to have been colonized by MDRO in the prior 6 months, and had longer LoS compared to patients with infection where no MDRO was recovered. Patients with MDRO infections were also more likely to be admitted for emergency surgery (28.7% vs 21.7%, *P* = 0.011) and have ICU-acquired infections (66.3% vs 32.6%, *P* < 0.0001). They also had higher SOFA scores at admission and were more likely to have a diagnosis of septic shock at enrollment (Table 2).

**TABLE 2:** Characteristics of patients by MDRO infection status^*c*^

Characteristic	Infected patients with no MDRO recovered^*a*^ *N* = 660 (70.3%)	Patients with MDRO infection *N* = 273 (29.3%)	*P* value
Age (years)	61.0 [43–72]	56.0 [39–78]	0.019
Male gender	414 (62.7%)	172 (63.0%)	0.937
Risk factors			
Respiratory disease	122 (18.5%)	29 (14.4%)	0.131
Obesity	163 (24.7%)	70 (25.8%)	0.717
Diabetes	154 (23.3%)	68 (24.9%)	0.607
Cardiovascular disease	45 (6.8%)	21 (7.8%)	0.615
Chronic liver disease	27 (4.1%)	10 (3.7%)	0.773
Chronic renal disease	71 (10.8%)	34 (12.5%)	0.444
Immunosuppression	50 (7.6%)	24 (8.9%)	0.512
Bone marrow transplantation	4 (0.6%)	1 (0.4%)	0.548
Solid organ transplantation	8 (1.2%)	8 (3.0%)	0.064
Hematology-oncology diagnosis	30 (4.6%)	14 (5.2%)	0.685
Chemotherapy in the previous 6 months	48 (7.3%)	2 (3.7%)	0.051
Human immunodeficiency virus positive	20 (3.0%)	12 (4.4%)	0.290
Alcohol use disorder	85 (12.9%)	36 (13.3%)	0.874
Smoker	211 (32.0%)	79 (29.2%)	0.399
Hospital admission in the prior 6 months	217 (32.9%)	103 (37.7%)	0.156
Use of antibiotics in the prior 6 months	237 (36.0%)	106 (38.8%)	0.409
MDRO colonization in the prior 6 months	33 (5.0%)	34 (12.6%)	<0.0001
Days from hospital admission to diagnosis of infection	3.0 [0–10]	12.0 [4–30]	<0.0001
Days from ICU admission to enrollment	6.0 [3–13]	16.0 [9–31]	<0.0001
Colonization by carbapenemase-producing Enterobacterales during ICU stay^*b*^	150/623 (24.1%)	118/252 (46.8%)	<0.0001
Location of infection acquisition			<0.0001
Community	282 (44.3%)	33 (12.2%)	
ICU	215 (32.6%)	181 (66.3%)	
Hospital non-ICU	112 (17.6%)	36 (13.3%)	
Long-term care	27 (4.3%)	20 (7.4%)	
Data on admission			
APACHE II score	16.0 [12–23]	17.0 [12–23]	0.178
SOFA score at admission	5.0 [3–8]	6.0 [3–9]	0.018
Type of admission			0.011
Medical	467 (70.8%)	165 (60.7%)	
Elective surgery	50 (7.6%)	29 (10.7%)	
Emergency surgery	143 (21.7%)	78 (28.7%)	
Trauma admission	78 (11.9%)	42 (15.4%)	0.143
Clinical status at enrollment			<0.0001
No infection	–^*d*^	–	
Infection without sepsis	314 (47.6%)	131 (49.1%)	
Probable sepsis	119 (18.0%)	14 (4.6%)	
Sepsis	107 (16.2%)	82 (29.8%)	
Septic shock	120 (18.2%)	46 (16.3%)	
SOFA score at enrollment	4.0 [2–7]	4.0 [2–7]	0.247
Length of ICU stay (days)	16.0 [8–33]	30.0 [17–35]	<0.0001

^*a*^ This group includes patients with culture-confirmed infections with non-MDRO and patients with probable infection (no positive culture).

^*b*^ Colonization by CPE refers to detection of CPE in a rectal swab during the incident hospital admission.

^*c*^ APACHE II: Acute Physiologic and Chronic Health Evaluation.

^*d*^ –, not applicable.

The risk factors independently associated with MDRO infection in the multivariable model included admission for emergency surgery, previous hospital admission in the last 6 months, CPE colonization during the incident hospitalization, LoS at enrollment, and hospital where the ICU was located (Table S3).

**Fig 2:**
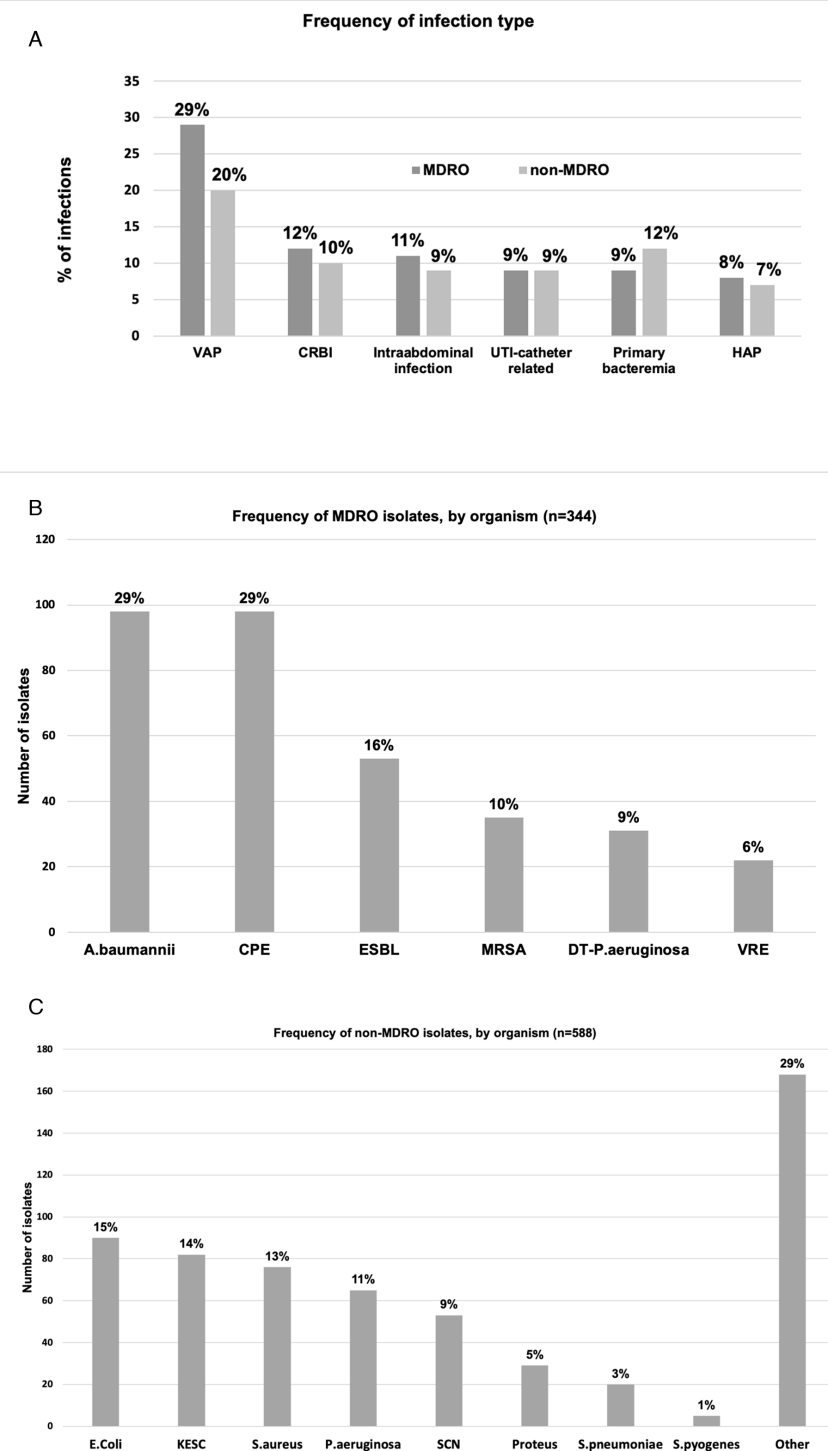
Localization of infections and microorganisms isolated. (A) VAP, ventilator-associated pneumonia; CRBSI, catheter-related bloodstream infection; IA, intra-abdominal infection; CR-UTI, catheter-related urinary tract infection; PBI, primary bacteremia; HAP, hospital-acquired pneumonia. (B) CRAB, carbapenemase-producing *Acinetobacter baumannii*; CPE, carbapenemase-producing Enterobacterales; ESBL, extended spectrum β-lactamase-producing microorganisms; MRSA, methicillin-resistant *Staphylococcus aureus*; DT-PAE, difficult-to-treat *Pseudomonas aeruginosa*; VRE, vancomycin-resistant Enterococci. (C) *E. coli, Escherichia coli*; KESC, group including *Klebsiella, Enterobacter, Serratia*, and *Citrobacter; S. aureus, Staphylococcus aureus; P. aeruginosa, Pseudomonas aeruginosa;* SCN*, Staphylococcus* coagulase-negative*; S. pneumoniae, Streptococcus pneumoniae; S. pyogenes, Streptococcus pyogenes.*

The most frequent types of infection where MDROs were recovered were ventilator-associated pneumonia (VAP) (100/344; 29.1%), catheter-related bloodstream infection (CRBSI) (40/344, 11.6%), catheter-related UTI (37/344, 10.8%), intra-abdominal infection, primary bacteremia (32/344, 9.3% each), and hospital-acquired, non-ventilator-associated pneumonia (HAP) (28/344, 8.1%). For non-MDRO infections, VAP was also the most common infection type (119/588, 20.2%), followed by primary bacteremia (68/588, 11.6%), CRBSI (56/588, 9.5%), intra-abdominal infection, and catheter-related UTI (52/588, 8.8% each), and HAP (40/588, 6.8%) (Fig. 2A; Table S4).

The most common MDROs isolated were CRAB and CPE (98/344, 28.5% each). MBL were the most common mechanism of resistance reported among MDRO. The most predominant non-MDROs were *E. coli* (90/588, 15.3%) and the KESC group (82/588, 13.9%) (Fig. 2B and C; Tables S4 and S5).

### Prevalence and determinants of CPE colonization

CPE colonization was detected through rectal swabs taken during the incident hospitalization; 420/1,696 (24.7%) patients had a positive rectal swab with CPE. CPE colonization was more frequent in patients with infection compared to those without infection (268/875 [30.6%] vs 152/821 [18.5%], *P* < 0.001) (Table S2). The prevalence of CPE colonization was higher in patients with MDRO infections compared to patients without recovery of an MDRO (118/252 [46.8%] vs 150/623 [24.1%], *P* < 0.001) (Table 2). Of the 420 patients colonized with CPE, 84 (20.0%) had subsequent CPE infections.

### Mortality

Four hundred eighty-seven of the 1,799 patients (27.1%) died prior to ICU discharge. Mortality (28 days) was 23.2% (417/1,799 patients). Among patients with non-MDRO culture-confirmed infections, ICU mortality was 27.9% (91/326). ICU mortality among patients with MDRO infection was 38.8% (106/273). For patients with probable infections, ICU mortality was 35.6% (113/334) (*P* = 0.013).

In bivariate analysis, non-survivors were significantly more likely to be older and have prior diagnoses of respiratory disease, chronic liver disease, hematology-oncology diagnosis, and human immunodeficiency virus infection. They were also more likely to have had a hospital admission in the prior 6 months and longer LoS during the incident hospitalization (Table 3). Non-survivors were sicker on admission than survivors, with significantly higher admission APACHE II and SOFA scores as well as higher SOFA scores at enrollment. Non-survivors were more likely to have been admitted for medical (vs surgical) reasons, had lengthier LoS at enrollment, and a higher prevalence of sepsis and septic shock. They were more likely to be colonized by CPE and have MDRO infections. Interestingly, there was also an association between non-survivors and probable or possible infection status (i.e., no positive culture returned). Hospital-acquired pneumonia and primary bacteremia were significantly more frequent in non-survivors than in survivors, and difficult-to-treat *P. aeruginosa* was the only organism that differed significantly between the subgroups.

**TABLE 3:** Characteristics of enrolled patients by survivor status^*d*^

Characteristic	Survivors *n* = 1,312 (72.9%)	Non-survivors *N* = 487 (27.1%)	*P* value
Age	59.0 [42–70]	62.0 [46–73]	<0.001
Male gender	804 (61.3%)	314 (64.5%)	0.214
Risk factors and comorbidities			
Respiratory disease	187 (14.3%)	89 (18.3)	0.038
Obesity	293 (22.3%)	119 (24.4%)	0.825
Diabetes	311 (22.5%)	101 (24.2%)	0.346
Alcohol-related problem	134 (10.2%)	60 (12.3)	0.210
Smoking habit	414 (30%)	128 (30.7%)	0.786
Cardiovascular disease	115 (8.8%)	32 (6.6%)	0.129
Chronic liver disease	34 (2.6%)	25 (5.1%)	0.007
Chronic renal disease	128 (9.8%)	59 (12.1%)	0.147
Immunosuppression	99 (7.6%)	46 (9.5%)	0.191
Bone marrow transplantation	6 (0.5%)	4 (0.8%)	0.356
Solid organ transplantation	18 (1.4%)	12 (2.5%)	0.109
Hematology-oncology diagnosis	51 (3.9%)	35 (7.2%)	0.004
Chemotherapy in the previous 6 months	70 (5.4%)	30 (6.2%)	0.504
Human immunodeficiency virus positive	25 (1.9%)	20 (4.1%)	0.008
Hospital admission in the prior 6 months	391 (29.8%)	173 (35.5%)	0.002
Use of antibiotics in the prior 6 months	401 (31.0%)	168 (34.5)	0.113
MDRO colonization in the prior 6 months	85 (6.5%)	35 (7.2%)	0.654
Days from ICU admission to enrollment	6 [2–17]	9 [3–20]	0.003
Colonization by carbapenemase-producing Enterobacterales^*a*^	283 (22.8%)	137 (30.2%)	0.002
Location of infection acquisition			0.969
Community	218 (34.9%)	97 (34.4%)	
ICU	274 (43.9%)	122 (43.3%)	
Hospital non-ICU	101 (16.2%)	47 (16.2%)	
Long-term care	31 (5.0%)	16 (5.7%)	
Data on admission			
APACHE II score	14 [10–19]	17 [12–23]	<0.001
SOFA score at admission	4 [2–7]	5 [3–8]	<0.001
Type of admission			<0.001
Medical	827 (63.1%)	349 (71.7%)	
Elective surgery	217 (16.6%)	49 (10.1%)	
Emergency surgery	267 (20.4%)	89 (18.3%)	
Trauma admission	195 (14.9%)	62 (12.7%)	0.246
Clinical status at enrollment			<0.001
No infection	693 (52.8%)	193 (40.0%)	
Infection without sepsis	342 (26.1%)	106 (21.8%)	
Sepsis	203 (15.4%)	105 (21.6%)	
Septic shock	74 (5.7%)	83 (17.0%)	
SOFA score at enrollment	2 [1–5]	5 [3–8]	<0.001
Infection status			0.013
Isolation of non-MDRO	251 (18.1%)	75 (18.0%)	
Isolation of MDRO	167 (13.4%)	91 (21.8%)	
Probable or possible infection (no organism recovered)	227 (16.4%)	107 (25.7%)	
Types of infection			
Community-acquired pneumonia	97 (7.4%)	44 (9.0%)	0.250
Hospital-acquired pneumonia	51 (3.9%)	44 (9.3%)	<0.001
Ventilator-associated pneumonia	138 (10.6%)	58 (11.9%)	0.400
Catheter-associated blood stream infection	59 (4.5%)	24 (4.9%)	0.698
Primary bacteremia	47 (3.6%)	28 (5.8%)	0.041
Cardiovascular infection	5 (0.4%)	4 (0.8%)	0.240
Community-acquired urinary tract infection	30 (2.3%)	12 (2.4%)	0.825
Catheter-associated urinary tract infection	43 (3.3%)	25 (5.1%)	0.067
Intra-abdominal infection	85 (6.5%)	37 (7.6%)	0.402
Surgical site infections	30 (2.3%)	10 (2.1%)	0.766
Hospital-acquired meningitis	28 (2.1%)	5 (1.0%)	0.120
Skin and soft tissue infection	35 (2.7%)	16 (3.3%)	0.483
Bone and joint infection	17 (1.3%)	3 (0.6%)	0.222
Gynecological and obstetric infection	7 (0.5%)	0 (0.0%)	0.145
Other	25 (1.9%)	10 (2.1%)	0.840
Principal microorganisms isolated			
*A. baumannii*	59 (4.5%)	32 (6.6%)	0.075
Difficult-to-treat *P. aeruginosa*	12 (1.2%)	14 (2.9%)	0.015
Carbapenemase-producing Enterobacterales	64 (4.9%)	32 (6.6%)	0.156
Extended spectrum-producing β-lactamase organisms	49 (3.7%)	17 (3.5%)	0.807
Methicillin-resistant *S. aureus*	23 (1.8%)	12 (2.5%)	0.332
Vancomycin-resistant *Enterococcus*	16 (1.2%)	5 (1.0%)	0.735
Other outcomes			
ICU length of stay (days)	15 [5–39]	22 [12–41]	<0.001
Antimicrobial use^*b*^			
By therapeutic approach (empiric vs directed therapy)			0.331
Empiric	310 (51.7%)	168 (55.1%)	
Directed	290 (48.3%)	137 (44.9%)	
By concordance with antimicrobial susceptibility testing results			0.180
Adequate	570 (90.3%)	244 (93.1%)	
Inadequate	61 (9.7%)	18 (6.9%)	
By type of antimicrobial			0.844
New	41 (6.5%)	16 (6.2%)	
Traditional	589 (93.5%)	244 (93.8%)	
By AWaRe classification^*c*^			0.770
Access	245 (38.5%)	95 (36.1%)	
Watch	268 (42.1%)	117 (44.5%)	
Reserve	124 (19.5%)	51 (19.4%)	
Characteristics of hospitals			
Management modality			<0.001
Public	682 (52.5%)	299 (61.9%)	
Private hospitals	616 (47.5%)	184 (38.1%)	
Presence of infection prevention and control committee	1252 (96.5%)	475 (98.6%)	0.067
Presence of antimicrobial stewardship program	744 (57.3%)	272 (56.4%)	0.724

^*a*^ Colonization by CPE refers to detection of CPE in a rectal swab during the incident hospital admission.

^*b*^ For antimicrobial use, the denominator is only patients who had an infection.

^*c*^ Classification of antimicrobials of the World Health Organization Expert Committee on Selection and Use of Essential Medicines (REF).

^*d*^ APACHE II: Acute Physiologic and Chronic Health Evaluation.

There were no differences among the different categories of antimicrobial use between survivors and non-survivors, and the only hospital-level variable that was significantly more frequent in non-survivors was admission to a public hospital (Table 3).

### Predictors of ICU mortality

In the multivariable analysis of risk factors for ICU mortality, age, infection status (infections with an MDRO recovered or with negative cultures, as compared to infection by non-MDRO), SOFA score at enrollment, and presence of hospital-acquired pneumonia were independently associated with higher risk of death prior to ICU discharge. In addition, the model demonstrated significant variations between hospitals in the individual risk of ICU death (var = 0.56, *P* < 0.001) (Table 4).

**TABLE 4:** Multivariable model of risk factors for in-ICU mortality

Mortality	OR	[95% CI]	*P* value	Variance (SE)	[95% CI]	*P* value^*a*^
Fixed effects						
Age	1.01	[1.00–1.02]	0.003			
Infection status						
Non-MDRO	Ref.^*b*^					
MDRO	1.65	[1.18–2.43]	0.012			
No microbiological isolation (probable or possible infection)	1.41	[0.97–2.04]	0.073			
SOFA score at enrollment	1.18	[1.13–1.23]	<0.0001			
Hospital-acquired pneumonia	1.84	[1.12–3.01]	0.016			
Random effects						
Hospital				0.56 (0.15)	[0.33–0.95]	0.0046

^*a*^ LR test comparing the model with ordinary logistic regression.

^*b*^ Reference.

## DISCUSSION

To the best of our knowledge, this is the first large-scale point-prevalence study of MDRO infections, CPE colonization, and mortality carried out exclusively in critical care units in a Latin-American country. We found a 15.1% prevalence of MDRO infections in the entire ICU population and a 24.7% CPE colonization prevalence in rectal swabs. In patients with definite infections, the proportion of MDRO infections was 45.6%. In-ICU mortality was significantly higher in patients with an MDRO recovered in culture, and patients with no organisms identified, compared to those with non-MDRO infections (38.8%, 35.6%, and 27.9%, respectively). Additionally, ICU LoS was significantly longer in patients with definite MDRO infections compared to patients with non-MDRO infections (30.0 [17–35] days vs16.0 [8–33], respectively).

Studies have described the burden of AMR in low- and middle-income countries (LMICs) over the last 30 years and modeled future impacts (9). According to a recent study published in the Lancet, by the year 2050 Latin-America and the Caribbean are projected to have 650,000 deaths associated with AMR annually with an AMR-associated mortality rate of 96.7/100.000 [95% UI 78–119], and 148,000 attributable deaths with an AMR-attributable mortality rate of 22.1/100.000 [95% UI 17.5–27.2]. These rates are higher than those projected for high-income countries in the same study: AMR-associated deaths 78.1/100.000 [95% UI 59.8–92.2] and AMR-attributable deaths 17/100.000 [95% UI 12.9–19.9] (9). Our findings, with almost half of the culture-positive infections in enrolled ICU patients yielding MDROs and the increased risk of mortality in patients with MDRO infections, appear to be in line with these projections. Our hope is that this local data can help raise awareness and advocate for more resources to prevent infections, strengthen antimicrobial stewardship programs, and improve access to new antimicrobials for MDROs. These remain key strategies to mitigate projected increases in AMR-related deaths in the future.

ICUs are the hospital units with the highest prevalence of healthcare-associated infections (HAIs) due to invasive procedures and the devices involved in critical care. These lead to increased antimicrobial consumption, selective pressure, emergence of MDROs, and their subsequent spread when infection control measures fail (14–16). The percentage of patients with suspected or proven infection (51.9%) was similar to the findings in the European Prevalence of Infection in Intensive Care Study (EPIC III), a 24-hour point-prevalence study conducted at 1,150 centers in 88 countries in September 2017, where 54% of ICU patients were documented as having an infection (4). Our study demonstrates that 32.8% of reported infections were HAIs, compared to 20.5% in other regions, but our estimates are comparable to findings from Brazil (16, 17). Ventilator-associated pneumonia was the primary site of infection as described in the EPIC III study and other studies focusing in LMICs, including those in Latin-America (4, 17, 18). Regarding the severity of infections, half presented severe clinical features: sepsis (33%) or septic shock (16.8%). While the proportion of patients with sepsis was similar to that of a previous study carried out in Argentina, the proportion of patients with septic shock markedly decreased (29% and 40%, respectively) (18). Differences in study design might account for these findings; however, more rapid recognition and treatment of infection and sepsis, as has been widely recommended, may have also contributed to this decrease (19).

Evidence regarding the proportion of patients colonized with CPE who go on to develop CPE infections is variable (20, 21). In a recent study, CPE colonization was not independently associated with mortality but with a higher risk of developing clinical infections and longer hospital stays (22). In our study, one out of five CPE colonized patients developed an infection caused by the same organism, similar to recently reported data from Brazil (21, 22).

Concerningly, the prevalence of CPE infections (28.5%) was significantly higher than that reported in other regions, for example, 6.6% in Europe (23–25). Regarding the mechanisms of resistance identified, there was a predominance of MBL, specific to the region (26). The main organisms recovered in VAP were *A. baumannii* and CPE, both identified as critical on the 2024 WHO bacterial priority pathogens list and associated with elevated mortality (27–29). The high prevalence of MDRO may have contributed to the high mortality rate observed in our patients; indeed, these MDROs have been identified as independent predictors of mortality in previous studies (5). However, *P. aeruginosa* was the only organism that differed significantly between survivors and non-survivors.

The prevalence of *Acinetobacter* spp. and CPE in device-associated infections highlights the urgent need to improve the implementation of antibiotic stewardship and infection prevention and control programs to prevent and mitigate the growing levels of AMR. Although 89% of the ICUs reported an infection prevention and control program in their hospitals, only half reported an antimicrobial stewardship program. It also remains unclear the extent to which these programs were functional and fully implemented in participating hospitals.

Compared to high-income countries, LMICs experience a higher burden of ICU-acquired infections and ICU mortality (33.6% vs <20% in HICs) (16, 30). The high ICU mortality rate described in this study aligns with previous data and helps narrow the knowledge gap for this region (8). MDRO infections have been identified as an independent predictor of mortality, which is one of the main findings of this study, underscoring the burden of AMR, particularly in limited resource settings (2, 4). Infection control and antimicrobial stewardship programs have demonstrated efficacy in preventing and containing AMR; however, the challenge in LMIC remains the full implementation of these programs across the healthcare spectrum.

Our study has several limitations. First, due to the cross-sectional design, we could not determine the causal direction of associations, apart from those associated with ICU mortality. Second, voluntary enrollment of hospitals in the study may have introduced a selection bias, since hospitals with infection prevention and control or antimicrobial stewardship programs might be more inclined to participate in the study. Third, point-prevalence studies are influenced by patient length of stay, potentially enrolling more patients with longer ICU stays, which could affect the evaluation of the risk factors for mortality. Fourth, only CPE colonization was measured; thus, we were unable to quantify the impact of colonization by other MDROs. Fifth, a large proportion of patients had probable or possible infections, so the prevalence of infections and MDRO mortality may have been underestimated. This factor was also an issue in the EPIC III study, in which 33% of infections were probable or possible, similar to our study’s finding of 35.8%. Finally, we did not have detailed information on patient antibiotic treatments and are thus unable to evaluate how access to new drugs (or lack of access) might have contributed to the high mortality observed. Regardless of these drawbacks, a clear strength of the study is the large number of participating ICUs, which allowed for the collection of data on infection patterns and mortality in a large and diverse group of patients across Argentina.

In conclusion, the PREV-AR study, which included a large number of ICUs from an LMIC, showed a high frequency of MDRO infections and CPE colonization, and there was an association with increased mortality and longer ICU stay. This study establishes a baseline for future studies in Argentina to track changes in AMR prevalence over time, and to implement and adapt antimicrobial stewardship programs and measures for prevention and control of MDRO infections.

## Data Availability

The data that support the findings of this study are available from the corresponding author, M.I.S., upon reasonable request, subject to approval by the relevant institutional review boards.

## SUPPLEMENTAL MATERIAL

Supplemental material aac.01426-24-s0001.docx Participating hospitals, Tables S1 to S6, manual of operations, and Appendices 1 to 4.

ASM does not own the copyrights to Supplemental Material that may be linked to, or accessed through, an article. The authors have granted ASM a non-exclusive, world-wide license to publish the Supplemental Material files. Please contact the corresponding author directly for reuse.

## References

[1] Antimicrobial Resistance Collaborators. 2023. The burden of antimicrobial resistance in the Americas in 2019: a cross-country systematic analysis. Lancet Reg Health Am 25:100561. doi:10.1016/j.lana.2023.100561 37727594 PMC10505822

[2] Ciapponi A, Bardach A, Sandoval MM, Palermo MC, Navarro E, Espinal C, Quirós R. 2023. Systematic review and meta-analysis of deaths attributable to antimicrobial resistance, Latin America. Emerg Infect Dis 29:2335–2344. doi:10.3201/eid2911.230753 37877573 PMC10617342

[3] Riley MM. 2021. Infection challenges in the critical care unit, an issue of critical care nursing clinics of North America. Elsevier Health Sciences, Netherlands.

[4] Vincent J-L, Sakr Y, Singer M, Martin-Loeches I, Machado FR, Marshall JC, Finfer S, Pelosi P, Brazzi L, Aditianingsih D, Timsit J-F, Du B, Wittebole X, Máca J, Kannan S, Gorordo-Delsol LA, De Waele JJ, Mehta Y, Bonten MJM, Khanna AK, Kollef M, Human M, Angus DC, EPIC III Investigators. 2020. Prevalence and outcomes of infection among patients in intensive care units in 2017. JAMA 323:1478–1487. doi:10.1001/jama.2020.2717 32207816 PMC7093816

[5] Araos R, Smith RM, Styczynski A, Sánchez F, Acevedo J, Maureira L, Paredes C, González M, Rivas L, Spencer-Sandino M, Peters A, Khan A, Sepulveda D, Wettig LR, Rioseco ML, Usedo P, Soto PR, Huidobro LA, Ferreccio C, Park BJ, Undurraga E, D’Agata EMC, Jara A, Munita JM. 2023. High burden of intestinal colonization with antimicrobial-resistant bacteria in Chile: an antibiotic resistance in communities and hospitals (ARCH) study. Clin Infect Dis 77:S75–S81. doi:10.1093/cid/ciad283 37406045 PMC10321693

[6] Vargas JM, Moreno Mochi MP, López CG, Alarcón JA, Acosta N, Soria K, Nuñez JM, Villafañe S, Ramacciotti J, del Campo R, Jure MA. 2022. Impacto de un programa de vigilancia activa y medidas de control de infecciones sobre la incidencia de bacilos gram negativos resistentes a carbapenems en una unidad de cuidados intensivos. Rev Arg Microbiol 54:134–142. doi:10.1016/j.ram.2021.03.003 34088536

[7] Ghosh S, Bornman C, Zafer MM. 2021. Antimicrobial resistance threats in the emerging COVID-19 pandemic: where do we stand? J Infect Public Health 14:555–560. doi:10.1016/j.jiph.2021.02.011 33848884 PMC7934675

[8] Martins APS, da Mata CPSM, Dos Santos UR, de Araújo CA, Leite EMM, de Carvalho LD, Vidigal PG, Vieira CD, Dos Santos-Key SG. 2023. Association between multidrug-resistant bacteria and outcomes in intensive care unit patients: a non-interventional study. Front Public Health 11:1297350. doi:10.3389/fpubh.2023.1297350 38259738 PMC10801015

[9] Naghavi M, Vollset SE, Ikuta KS, Swetschinski LR, Gray AP, Wool EE, Robles Aguilar G, Mestrovic T, Smith G, Han C, et al.. 2024. Global burden of bacterial antimicrobial resistance 1990–2021: a systematic analysis with forecasts to 2050. Lancet 404:1199–1226. doi:10.1016/S0140-6736(24)01867-1 39299261 PMC11718157

[10] Singer M, Deutschman CS, Seymour CW, Shankar-Hari M, Annane D, Bauer M, Bellomo R, Bernard GR, Chiche J-D, Coopersmith CM, Hotchkiss RS, Levy MM, Marshall JC, Martin GS, Opal SM, Rubenfeld GD, van der Poll T, Vincent J-L, Angus DC. 2016. The third international consensus definitions for sepsis and septic shock (Sepsis-3). JAMA 315:801–810. doi:10.1001/jama.2016.0287 26903338 PMC4968574

[11] Calandra T, Cohen J, International Sepsis Forum Definition of Infection in the ICU Consensus Conference. 2005. The international sepsis forum consensus conference on definitions of infection in the intensive care unit. Crit Care Med 33:1538–1548. doi:10.1097/01.CCM.0000168253.91200.83 16003060

[12] Paul M, Carrara E, Retamar P, Tängdén T, Bitterman R, Bonomo RA, de Waele J, Daikos GL, Akova M, Harbarth S, Pulcini C, Garnacho-Montero J, Seme K, Tumbarello M, Lindemann PC, Gandra S, Yu Y, Bassetti M, Mouton JW, Tacconelli E, Rodríguez-Baño J. 2022. European society of clinical microbiology and infectious diseases (ESCMID) guidelines for the treatment of infections caused by multidrug-resistant Gram-negative bacilli (endorsed by European society of intensive care medicine). Clin Microbiol Infect 28:521–547. doi:10.1016/j.cmi.2021.11.025 34923128

[13] World Health Organization. 2023. WHO AWaRe (access, watch, reserve) classification of antibiotics for evaluation and monitoring of use. Available from: https://www.who.int/publications/i/item/WHO-MHP-HPS-EML-2023.04. Retrieved 19 Mar 2024.

[14] European Centre for Disease Prevention and Control. 2023. Antimicrobial resistance in the EU/EEA (EARS-Net) - annual epidemiological report 2022. Stockholm: ECDC. Available from: https://www.ecdc.europa.eu/en/publications-data/surveillance-antimicrobial-resistance-europe-2022. Retrieved 19 Mar 2024.

[15] Fridkin SK. 2001. Increasing prevalence of antimicrobial resistance in intensive care units. Crit Care Med 29:N64–N68. doi:10.1097/00003246-200104001-00002 11292878

[16] European Centre for Disease Prevention and Control. 2024. Point prevalence survey of healthcare-associated infections and antimicrobial use in European acute care hospitals. Stockholm: ECDC. Available from: https://www.ecdc.europa.eu/en/publications-data/PPS-HAI-AMR-acute-care-europe-2022-2023. Retrieved 20 Mar 2024.

[17] Machado FR, Cavalcanti AB, Bozza FA, Ferreira EM, Angotti Carrara FS, Sousa JL, Caixeta N, Salomao R, Angus DC, Pontes Azevedo LC, SPREAD Investigators, Latin American Sepsis Institute Network. 2017. The epidemiology of sepsis in Brazilian intensive care units (the Sepsis PREvalence assessment database, SPREAD): an observational study. Lancet Infect Dis 17:1180–1189. doi:10.1016/S1473-3099(17)30322-5 28826588

[18] Estenssoro E, Loudet CI, Edul VSK, Osatnik J, Ríos FG, Vásquez DN, Pozo MO, Lattanzio B, Pálizas F, Klein F, Piezny D, Rubatto Birri PN, Tuhay G, Díaz A, Santamaría A, Zakalik G, Dubin A, investigators of the SATISEPSIS Group. 2019. Health inequities in the diagnosis and outcome of sepsis in Argentina: a prospective cohort study. Crit Care 23:250. doi:10.1186/s13054-019-2522-6 31288865 PMC6615149

[19] Evans L, Rhodes A, Alhazzani W, Antonelli M, Coopersmith CM, French C, Machado FR, Mcintyre L, Ostermann M, Prescott HC, et al.. 2021. Surviving sepsis campaign: international guidelines for management of sepsis and septic shock 2021. Intensive Care Med 47:1181–1247. doi:10.1007/s00134-021-06506-y 34599691 PMC8486643

[20] McConville TH, Sullivan SB, Gomez-Simmonds A, Whittier S, Uhlemann AC. 2017. Carbapenem-resistant Enterobacteriaceae colonization (CRE) and subsequent risk of infection and 90-day mortality in critically ill patients, an observational study. PLoS One 12:e0186195. doi:10.1371/journal.pone.0186195 29023567 PMC5638409

[21] Gomides MDA, Fontes AM de S, Silveira AOSM, Matoso DC, Ferreira AL, Sadoyama G. 2022. The importance of active surveillance of carbapenem-resistant Enterobacterales (CRE) in colonization rates in critically ill patients. PLoS One 17:e0262554. doi:10.1371/journal.pone.0262554 35051212 PMC8775193

[22] Hassoun-Kheir N, Hussien K, Karram M, Saffuri M, Badaan S, Peleg S, Aboelhega W, Warman S, Alon T, Pollak D, Szwarcwort Cohen M, Paul M. 2023. Clinical significance and burden of carbapenem-resistant Enterobacterales (CRE) colonization acquisition in hospitalized patients. Antimicrob Resist Infect Control 12:129. doi:10.1186/s13756-023-01323-y 37986092 PMC10658805

[23] Aleidan FAS, Alkhelaifi H, Alsenaid A, Alromaizan H, Alsalham F, Almutairi A, Alsulaiman K, Abdel Gadir AG. 2021. Incidence and risk factors of carbapenem-resistant Enterobacteriaceae infection in intensive care units: a matched case-control study. Expert Rev Anti Infect Ther 19:393–398. doi:10.1080/14787210.2020.1822736 32930620

[24] Budhram DR, Mac S, Bielecki JM, Patel SN, Sander B. 2020. Health outcomes attributable to carbapenemase-producing Enterobacteriaceae infections: a systematic review and meta-analysis. Infect Control Hosp Epidemiol 41:37–43. doi:10.1017/ice.2019.282 31637986

[25] European Centre for Disease Prevention and Control. 2020. Healthcare-associated infections acquired in intensive care units annual epidemiological report for 2020. Available from: https://www.ecdc.europa.eu/sites/default/files/documents/healthcare-associated-infections-intensive-care-units-annual-epidemiological-report-2020.pdf. Retrieved 21 Mar 2024.

[26] Mojica MF, Rossi MA, Vila AJ, Bonomo RA. 2022. The urgent need for metallo-β-lactamase inhibitors: an unattended global threat. Lancet Infect Dis 22:e28–e34. doi:10.1016/S1473-3099(20)30868-9 34246322 PMC8266270

[27] Paniagua-García M, Bravo-Ferrer JM, Pérez-Galera S, Kostyanev T, de Kraker MEA, Feifel J, Palacios-Baena ZR, Schotsman J, Cantón R, Daikos GL, et al.. 2024. Attributable mortality of infections caused by carbapenem-resistant Enterobacterales: results from a prospective, multinational case-control-control matched cohorts study (EURECA). Clin Microbiol Infect 30:223–230. doi:10.1016/j.cmi.2023.11.008 38267096

[28] WHO. 2024. WHO bacterial priority pathogens list, 2024: bacterial pathogens of public health importance to guide research, development and strategies to prevent and control antimicrobial resistance. Available from: https://iris.who.int/bitstream/handle/10665/376776/9789240093461-eng.pdf?sequence=1. Retrieved 21 Mar 2024.

[29] Lemos EV, de la Hoz FP, Einarson TR, McGhan WF, Quevedo E, Castañeda C, Kawai K. 2014. Carbapenem resistance and mortality in patients with Acinetobacter baumannii infection: systematic review and meta-analysis. Clin Microbiol Infect 20:416–423. doi:10.1111/1469-0691.12363 24131374

[30] Saharman YR, Karuniawati A, Severin JA, Verbrugh HA. 2021. Infections and antimicrobial resistance in intensive care units in lower-middle income countries: a scoping review. Antimicrob Resist Infect Control 10:22. doi:10.1186/s13756-020-00871-x 33514432 PMC7844809

