# Supplementary material for: Prevalence and mortality associated with multidrug-resistant infections in adult intensive care units in Argentina (PREV-AR)
Source: Antimicrob Agents Chemother. 2025 Jan 22;69(3):e01426-24. doi: 10.1128/aac.01426-24 (PMC11881575; doi:10.1128/aac.01426-24)
Supplement: Supplemental material — Participating hospitals, Tables S1 to S6, manual of operations, and Appendices 1 to 4. [file aac.01426-24-s0001.docx]

**Supplemental information**

**Participating hospitals (n=164)**

| Province | Hospital | Number of patients included |
| --- | --- | --- |
| Buenos Aires | Clínica Colón Mar del Plata | 10 |
|  | Clínica Olivos | 13 |
|  | Hospital Trauma y Emergencias Médicas Dr. Abete | 19 |
|  | Hospital A Eurnekian Ezeiza | 10 |
|  | Hospital del Bicentenario de Esteban Echeverría | 26 |
|  | Hospital Cuenca Alta Néstor Kirchner Cañuelas | 19 |
|  | Hospital Cetrangolo | 4 |
|  | Hospital Carlos F Macia Mar de Ajo | 2 |
|  | Hospital Dra. Cecilia Grierson de Guernica | 1 |
|  | Hospital Central de Pilar | 20 |
|  | Hospital Central de San Isidro Melchor Posse | 13 |
|  | Hospital El Cruce Néstor Kirchner | 15 |
|  | Hospital Emilio Ferreyra de Necochea | 4 |
|  | Hospital Eva Perón San Martin (ExCastex ) | 12 |
|  | Hospital Escardó de Tigre | 1 |
|  | Hospital Español de La Plata | 6 |
|  | Hospital Zonal General de Agudos Héroes de Malvinas | 8 |
|  | Hospital Zonal General de Agudos General Manuel Belgrano | 7 |
|  | Hospital Houssay | 5 |
|  | HIEAYC San Juan de Dios de La Plata | 8 |
|  | Hospital Interzonal General de Agudos Evita Pueblo | 6 |
|  | Hospital Interzonal General de Agudos Evita | 9 |
|  | Hospital Interzonal General de Agudos Gandulfo | 12 |
|  | Hospital Interzonal General de Agudos Junín | 5 |
|  | Hospital Interzonal General de Agudos Dr Oscar Alende | 14 |
|  | Hospital Interzonal General de Agudos Pte Perón de Avellaneda | 6 |
|  | Hospital Interzonal General de Agudos Dr. R. Rossi | 7 |
|  | Hospital Interzonal General de Agudos San Martín de La Plata | 16 |
|  | Hospital Interzonal General de Agudos San Roque Gonnet | 8 |
|  | Hospital Interzonal General de Agudos y Crónicos (HIEAYC) Alejandro Korn de Romero | 10 |
|  | Hospital Interzonal General de Agudos Paroissien de La Matanza | 9 |
|  | Hospital Interzonal General de Agudos Pedro Fiorito | 12 |
|  | Hospital Interzonal Gral. de Agudos San José de Pergamino | 5 |
|  | Hospital Italiano de La Plata | 25 |
|  | Hospital Italiano de San Justo | 26 |
|  | Hospital Julio F. Ramos | 3 |
|  | Hospital Larcade | 8 |
|  | Hospital Municipal de Chivilcoy | 1 |
|  | Hospital Municipal de Agudos Dr. Leónidas Lucero | 11 |
|  | Hospital de Morón | 7 |
|  | Hospital Municipal Presidente Derqui | 1 |
|  | Hospital Zonal General de Agudos Mi Pueblo UPA 11 Florencio Varela | 5 |
|  | Hospital Nacional Alejandro Posadas | 31 |
|  | Hospital Privado Nuestra Señora de la Merced | 22 |
|  | Hospital Privado Dr. Raul Matera | 3 |
|  | Hospital Petrona V de Cordero | 7 |
|  | Hospital de Quilmes | 8 |
|  | Hospital Ramon Santamarina | 7 |
|  | Hospital SAMIC Dr. René Favaloro | 6 |
|  | Hospital Sanguinetti | 4 |
|  | Hospital San Cayetano | 10 |
|  | Hospital Simplemente Evita | 11 |
|  | Hospital Universitario Austral | 16 |
|  | Hospital Zonal E F Erill (Escobar) | 6 |
|  | Hospital Zonal General de Agudos Dr. A. Balestrini | 9 |
|  | Hospital Zonal General de Agudos Dr. Arturo Oñativia | 7 |
|  | Hospital Zonal General de Agudos M. V. de Martinez | 8 |
|  | Hospital Zonal General de Agudos Mi Pueblo | 4 |
|  | Hospital Zonal General de Agudos Narciso López Lanús | 5 |
|  | Hospital Zonal de Rehabilitación El Dique | 7 |
|  | Instituto del Diagnostico de La Plata | 10 |
|  | Clínica IPENSA de La Plata | 13 |
|  | Sanatorio Las Lomas | 6 |
|  | Sanatorio San Lucas | 11 |
|  | Sanatorio Trinidad Ramos Mejía | 25 |
|  | AMEBPBA CABA | 6 |
| City of Buenos Aires | Clinica Bazterrica | 19 |
|  | CEMIC | 17 |
|  | Centro Gallego | 23 |
|  | CIAREC | 8 |
|  | Centro Médico Integral Fitz Roy | 9 |
|  | Fundacion Favaloro | 20 |
|  | Instituto Flemming | 12 |
|  | FLENI | 9 |
|  | Hospital Aeronáutico Central | 3 |
|  | Hospital Argerich | 29 |
|  | Hospital Dr. Abel Zubizarreta | 4 |
|  | Hospital Británico | 18 |
|  | Hospital Medico Policial Churruca Visca | 13 |
|  | Hospital de Clinicas | 17 |
|  | Hospital Fernández | 24 |
|  | Hospital Italiano Central | 73 |
|  | Hospital J M Penna | 8 |
|  | Hospital Muñiz | 18 |
|  | Hospital Naval | 13 |
|  | Hospital Pirovano | 9 |
|  | Hospital Vélez Sarsfield | 4 |
|  | Instituto Argentino de Diagnóstico y Tratamiento | 26 |
|  | IMAC | 12 |
|  | ITAC | 9 |
|  | Policlínico Bancario | 7 |
|  | Sanatorio Anchorena | 23 |
|  | Sanatorio Colegiales | 27 |
|  | Sanatorio Franchin | 9 |
|  | Sanatorio de Los Arcos | 17 |
|  | Sanatorio Mater Dei | 11 |
|  | Sanatorio San Camilo | 8 |
|  | Sanatorio de la Trinidad Mitre | 11 |
|  | Unidad Asistencial Dr. César Milstein | 9 |
| Catamarca | Hospital San Juan Bautista | 5 |
| Chubut | Clínica del Valle | 6 |
|  | Hospital Andrés Isola | 2 |
|  | Hospital Regional de Comodoro Rivadavia |  |
|  | Hospital Regional V Sanguinetti de Comodoro Rivadavia | 4 |
|  | Hospital Santa Teresita | 1 |
|  | Hospital Zonal Alvear de Comodoro Rivadavia | 3 |
|  | Hospital Zonal Esquel | 6 |
|  | Hospital Zonal de Trelew Dr. Adolfo Margara | 7 |
| Córdoba | Hospital Nacional de Clínicas | 10 |
|  | Hospital Nuestra Señora de la Misericordia | 14 |
| Corrientes | Clínica del Sol Corrientes | 5 |
|  | Instituto de Cardiología de Corrientes | 12 |
| Entre Rios | CDI Hospital de la Baxada Teresa Ratto | 8 |
|  | Hospital Centenario de Gualeguaychú | 5 |
|  | Hospital San Martín Paraná Entre Ríos | 13 |
|  | Instituto Médico Quirúrgico Garat | 8 |
| Formosa | Hospital Interdistrital Evita de la ciudad de Formosa. | 15 |
| Jujuy | Hospital Pablo Soria | 9 |
| La Pampa | Hospital Favaloro (ex Hospital Lucio Molas) | 17 |
|  | Hospital Gobernador Centeno, Gral Pico | 7 |
| La Rioja | Hospital Regional Dr. Enrique Vera Barros | 6 |
| Mendoza | Hospital Alfredo Italo Perrupato | 9 |
|  | Hospital el Carmen de Mendoza | 17 |
|  | Hospital Central de Mendoza | 5 |
|  | Hospital Italiano de Mendoza | 5 |
|  | Hospital Lagomaggiore | 11 |
| Misiones | Hospital Escuela de Agudos Dr. Ramón Madariaga | 26 |
|  | Hospital Nivel III de Obera | 9 |
| Neuquén | CMIC Neuquén | 6 |
| Rïo Negro | Asociación Española de Socorros Mutuos | 7 |
|  | Clinica Viedma | 4 |
|  | Hospital Francisco López Lima de General Roca, Rio Negro. | 4 |
|  | Hospital Pedro Moguillansky | 3 |
|  | Hospital Zatti Viedma | 7 |
|  | Hospital Zonal de Bariloche Ramón Carrillo | 7 |
|  | Policlinico Modelo de Cipolletti | 17 |
| Salta | Hospital Arturo Oñativia de Salta | 10 |
|  | Hospital Público Materno Infantil | 12 |
|  | Hospital Sr. del Milagro | 9 |
| San Luis | Hospital Central Ramón Carrillo | 10 |
|  | Hospital Regional J.D. Perón Villa Mercedes | 4 |
| Santa Cruz | Hospital Regional Rio Gallegos | 3 |
| Santa Fe | Hospital General Especializado en Oncología J. B. Iturraspe | 10 |
|  | Hospital Italiano Rosario | 8 |
|  | Hospital Dr. José María Cullen | 18 |
|  | Hospital Provincial del Centenario | 11 |
|  | Hospital Privado de Rosario | 5 |
|  | Samco Jaime Ferre | 5 |
|  | Sanatorio Italiano Centro | 10 |
|  | Sanatorio Médico Quirúrgico Santa Fe | 9 |
|  | Sanatorio Parque | 14 |
| Tierra del Fuego | Clínica CEMEP Tierra del Fuego | 11 |
|  | Hospital Regional Rio Grande Tierra del Fuego | 5 |
|  | Sanatorio San Jorge Ushuaia | 8 |
| Tucumán | Hospital de Clínicas Pte Nicolás Avellaneda | 21 |
|  | Hospital Centro de Salud Zenón Santillán. San Miguel de Tucumán | 10 |
|  | Hospital Eva Perón Banda de Río Sali, Tucumán | 12 |
|  | Hospital Néstor Kirchner | 7 |
|  | Hospital Padilla de Tucumán | 33 |
|  | Instituto de Maternidad y Ginecología Nuestra Señora de la Mercedes | 3 |
|  | Sanatorio 9 de Julio | 10 |
|  | Sanatorio Modelo de Tucumán | 9 |
|  | Sanatorio Rivadavia | 25 |
|  | Sanatorio del Sur - San Miguel de Tucumán | 14 |

Table S1 . MDRO included in the study.

| **MDRO** | **DEFINITION** |
| --- | --- |
| Extended spectrum β-lactamase producing microorganisms (ESBL) | Isolation resistant to third and fourth generation cephalosporins; indicates resistance to aminopenicillins,ureidopenicillins, carboxypenicillins, first and second generation cephalosporins (excluding cephamycins), and monobactams. The presence of the enzyme in isolates can be confirmed by phenotypic methods: disk diffusion and automated systems (Phoenix, VITEK) or genotypic methods: PCR for identifying Extended-Spectrum Beta-Lactamase |
| Carbapenemase-producing enterobacterales (CPE), | Carbapenem-resistant isolates in which the presence of the carbapenemase enzyme was confirmed, according to the available diagnostic methods.  Genotypic methods: detection of different genes: KPC, OXA, NDM, VIM, IMP.  Phenotypic methods: disk diffusion methods and synergy evaluation, immunochromatographic methods, colorimetric methods, and microbiological methods. |
| Difficult-to treat *P. aeruginosa* (DTR-PAE) | DTR is defined as *P. aeruginosa* exhibiting non-susceptibility to all of the following: piperacillin-tazobactam, ceftazidime, cefepime, aztreonam, meropenem, imipenem-cilastatin, ciprofloxacin, and levofloxacin. |
| Carbapenem-resistant *A.baumannii* (CRAB) | Carbapenem-resistant *Acinetobacter spp* isolates (*Acinetobacter baumannii/calcoaceticus complex, Acinetobacter baumannii*, and other *Acinetobacter* species).  94.7% of carbapenem resistance in CRAB (carbapenem-resistant Acinetobacter baumannii) is due to OXA-23 oxacillinase, 4.6% to NDM, and 0.7% to OXA + NDM.* |
| Vancomycin-resistant enterococci (VRE) | *Enterococcus faecium* and *Enterococcus faecalis*, that have developed resistance to the antibiotic vancomycin mainly through the alteration of the target site for vancomycin. |
| Methicillin-resistant *S. aureus* (MRSA) | *S. aureus* with resistance to all beta-lactams and their combination with beta-lactamase inhibitors (except ceftaroline and ceftobiprole) can be detected using genotypic methods: PCR (detection of the MecA gene) or phenotypic methods: immunochromatographic, according to oxacillin interpretation. |

*WHONET Report, Argentina 2023: <http://antimicrobianos.com.ar/wp-content/uploads/2024/05/Vigilancia-Nacional-de-la-Resistencia-a-los-Antimicrobianos-Red-WHONET-Argentina-Tendencia-2013-2023-parcial.pdf>

Table S2. Characteristics of enrolled patients by infection status.

| Variable | All patients  1799 (100%) | Patients without infection  N=866 (48.1%) | Patients with  infection  N=933 (51.9%) | p  value |
| --- | --- | --- | --- | --- |
| Age (years) | 59.0 [43-71] | 59.0 [43-72] | 59.0 [43-71] | 0.366 |
| Male gender | 1118 (62.1%) | 533 (61.5%) | 585 (60.6%) | 0.572 |
| Risk factors |  |  |  |  |
| Respiratory disease | 276 (15.3%) | 116 (13.4%) | 160 (17.1%) | 0.025 |
| Obesity | 401 (22.3%) | 73 (8.4%) | 121 (13.0%) | 0.002 |
| Diabetes | 412 (22.9%) | 190 (21.9%) | 222 (23.8%) | 0.337 |
| Cardiovascular disease | 147 (8.2%) | 81 (9.4%) | 66 (7.1%) | 0.082 |
| Chronic liver disease | 59 (3.3%) | 22 (2.5%) | 37 (4.0%) | 0.087 |
| Chronic renal disease | 187 (10.4%) | 82 (9.5%) | 105 (11.3%) | 0.206 |
| Immunosuppression | 145 (8.1%) | 71 (8.1%) | 74 (7.9%) | 0.857 |
| Bone marrow transplantation | 10 (0.6%) | 5 (0.6%) | 5 (0.5%) | 0.913 |
| Solid organ transplantation | 30 (1.7%) | 14 (1.6%) | 16 (1.7%) | 0.864 |
| Hematology-oncology diagnosis | 86 (4.8%) | 42 (4.8%) | 44 (4.7%) | 0.911 |
| Chemotherapy in the prior 6 months | 100 (5.6%) | 42 (4.8%) | 58 (6.2%) | 0.200 |
| Human immunodeficiency virus postive | 45 (2.5%) | 13 (1.5%) | 32 (3.4%) | 0.008 |
| Alcohol use disorder | 194 (10.8%) | 73 (8.4%) | 121 (13.0%) | 0.002 |
| Smoker | 542 (30.1%) | 253 (29.2%) | 289 (31.0%) | 0.382 |
| Hospital admission in the prior 6 months | 564 (31.4%) | 244 (28.2%) | 320 (34.3%) | 0.005 |
| Use of antibiotics in the prior 6 months | 569 (31.6%) | 226 (26.1%) | 343 (36.7%) | <0.0001 |
| Colonization by MDRO in the prior 6 months | 120 (6.7%) | 53 (6.1%) | 67 (7.2%) | 0.358 |
| Days from ICU admission to enrollment | 7.0 [2-18] | 5.0 [2-18] | 8.0 [3-18] | <0.0001 |
| Colonization by carbapenemase-producing enterobacterales* | 420 (23.3%) | 152/821 (18.5%) | 268/875 (30.6%) | <0.0001 |
| Location of infection acquisition |  |  |  |  |
| Community | 315 (17.5%) |  | 315 (33.8%) |  |
| ICU | 396 (22.0%) |  | 396 (42.4%) |  |
| Hospital non-ICU | 148 (8.2%) |  | 148 (15.9%) |  |
| Long-term care | 47 (2.6%) |  | 47 (5.0%) |  |
| Healthcare-associated infections** | 591 (32.8%) |  | 591 (63.3%) |  |
| **Data on Admission** |  |  |  |  |
| APACHE II score | 15.0 [10-21] | 13.0 [9-19] | 16.0 [12-22] | <0.0001 |
| SOFA score on admission | 4.0 [2-8] | 3.0 [1-6] | 6.0 [3-8] | <0.0001 |
| SOFA score at enrollment | 3.0 [1-6] | 2.0 [1-4] | 4 .0[2-7] | <0.0001 |
| Type of admission |  |  |  | <0.0001 |
| Medical | 1176 (65.4%) | 544 (62.8%) | 632 (67.7%) |  |
| Elective surgery | 266 (14.8%) | 187 (20.0%) | 79 (8.4%) |  |
| Emergency surgery | 356 (19.8%) | 136 (14.6%) | 220 (23.6%) |  |
| Trauma admission | 257 (14.3%) | 138 (15.9%) | 119 (12.8%) | 0.059 |
| **Clinical status at enrollment** |  |  |  |  |
| No infection | 866 (48.1%) | 866 (100%) | - |  |
| Infection without sepsis | 448 (24.9.0%) | - | 448 (48.0%) |  |
| Sepsis | 308 (17.1%) | - | 308 (33.0%) |  |
| Septic shock | 157 (8.7%) | - | 157 (16.8%) |  |
| **Infection status** |  |  |  |  |
| Isolation of non-MDRO | 326 (18.1%) |  | 326 (34.9%) |  |
| Isolation of MDRO | 273 (15.2%) |  | 273 (29.3%) |  |
| Probable or possible infection (no organism recovered) | 334 (18.6%) |  | 334 (35.8%) |  |

*Colonization by carbapenemase-producing Enterobacterales (CPE) refers to detection of CPE in a rectal swab during the incident hospital admission

** Healthcare-associated infections represent the sum of infections acquired in the ICU, in hospital settings other than the ICU, and in the 3rd level.

Abbreviations. MDRO: multidrug-resistant microorganisms; ICU: Intensive Care Unit, APACHE II: Acute Physiologic and Chronic Health Evaluation; SOFA: Sepsis-related Organ Failure Assessment.

Table S3. Multivariable model of risk factors for MDRO infection.

| Infection by MDRO | OR | [95%  Conf.Interval] | P  value | Variance  (se) | [95%  Conf.Interval] | P value* |
| --- | --- | --- | --- | --- | --- | --- |
| Fixed effects |  |  |  |  |  |  |
| Admission to a hospital in the previous 6 months. | 1.56 | [1.08-2.24] | 0.017 |  |  |  |
| Carbapenemase-producing Enterobacteriaceae colonization | 2.86 | [1.96-4.20] | <0.0001 |  |  |  |
| Days from ICU admission to enrollment | 1.02 | [1.01-1.03] | <0.0001 |  |  |  |
| Type of admission |  |  |  |  |  |  |
| Medical | Ref. |  |  |  |  |  |
| Elective surgery | 1.73 | [1.10-3.13] | 0.072 |  |  |  |
| Emergency surgery | 1.56 | [1.04-2.32] | 0.031 |  |  |  |
| Random effects |  |  |  |  |  |  |
| Hospital |  |  |  | 0.70  (0.15) | [0.46-1.07] | 0.0001 |

*LR test comparing the model with ordinary logistic regression.

Table S4. Sites of infections and organisms isolated.

| **Multi-drug resistant**  **organisms** | | | | | | | | **Non multi-drug resistant**  **organisms** | | | | | | | | | |
| --- | --- | --- | --- | --- | --- | --- | --- | --- | --- | --- | --- | --- | --- | --- | --- | --- | --- |
| **Site of infection** | **Total number** | ***A.baumanii*** | **DT-*P.aeruginosa*** | **CPE** | **ESBL** | **VRE** | **MRSA** | **Total number** | ***S.pneumoniae*** | ***P.aeruginosa*** | ***S.aureus*** | ***E.coli*** | ***S.coagulase negative*** | ***Proteus sp.*** | ***KESC*** | ***S.pyogenes*** | **Other** |
| Community-acquired pneumonia | 6  (1.7%) | 0  (0.0%) | 0  (0.0%) | 1  (1.0%) | 2  (3.8%) | 0  (0.0%) | 2 (5.7%) | 42 (7.1%) | 10  (50.0%) | 3  (4.6%) | 9  (11.8%) | 2 (2.2%) | 3  (5.7%) | 0  (0.0%) | 3  (3.7%) | 0  (0.0%) | 12  (7.1%) |
| Hospital-acquired pneumonia | 28  (8.1%) | 10  (10.2%) | 2  (2.0%) | 3  (3.1%) | 5  (9.4%) | 2  (9.1%) | 2  (5.7%) | 40 (6.8%) | 2  (10.0%) | 7  (10.8%) | 5  (6.6%) | 4 (4.4%) | 1  (1.9%) | 2  (6.9%) | 8  (9.8%) | 0  (0.0%) | 11  (6.5%) |
| Ventilator-associated pneumonia | 100 (29.1%) | 50  (51.0%) | 11  (35.5%) | 26  (26.5%) | 11  (20.8%) | 3  (13.6%) | 9 (25.7%) | 119 (20.2%) | 3  (15.0%) | 27  (41.5%) | 24 (31.6%) | 7 (7.7%) | 4  (7.5%) | 8  (27.6%) | 17 (20.7%) | 0  (0.0%) | 29  (17.3%) |
| Catheter-related bloodstream infection | 40  (11.6%) | 0  (0.0%) | 2  (6.5%) | 12  (12.2%) | 3  (5.7%) | 3  (13.6%) | 7 (20.0%) | 56 (9.5%) | 0  (0.0%) | 4  (6.2%) | 12 (15.8%) | 5 (5.5%) | 9  (17.0%) | 4  (13.8%) | 8 (9.8%) | 1  (20.0%) | 13  (7.7%) |
| Primary bacteremia | 32  (9.3%) | 9  (9.2%) | 3  (9.8%) | 10  (10.2%) | 4  (7.5%) | 2  (9.1%) | 3 (8.8%) | 68 (11.6%) | 4  (20.0%) | 5  (7.7%) | 7  (9.2%) | 8 (8.8%) | 11  (20.8%) | 3  (10.3%) | 11 (13.4%) | 0  (0.0%) | 19  (11.3%) |
| Clostridium difficile diarrhea | 0  (0.0%) | 0  (0.0%) | 0  (0.0%) | 0  (0.0%) | 0  (0.0%) | 0  (0.0%) | 0 (0.0%) | 14 (2.4%) | 0  (0.0%) | 0  (0.0%) | 0  (0.0%) | 1 (1.1%) | 0  (0.0%) | 0  (0.0%) | 2  (2.4%) | 0  (0.0%) | 11  (6.5%) |
| Cardiovascular infections | 1  (0.3%) | 0  (0.0%) | 0  (0.0%) | 0  (0.0%) | 0  (0.0%) | 0  (0.0%) | 1 (2.9%) | 6 (1.0%) | 0  (0.0%) | 0  (0.0%) | 2  (2.6%) | 0 (0.0%) | 0  (0.0%) | 0  (0.0%) | 0  (0.0%) | 0  (0.0%) | 4  (2.4%) |
| Gynecological and obstetric | 1  (0.3%) | 1  (1.0%) | 0  (0.0%) | 0  (0.0%) | 0  (0.0%) | 0  (0.0%) | 0 (0.0%) | 4 (0.6%) | 0  (0.0%) | 0  (0.0%) | 0  (0.0%) | 2 (2.2%) | 0  (0.0%) | 0  (0.0%) | 0  (0.0%) | 0  (0.0%) | 2  (1.2%) |
| UTI-community | 5  (1.5%) | 0  (0.0%) | 0  (0.0%) | 0  (0.0%) | 5  (9.4%) | 0  (0.0%) | 0 (0.0%) | 33 (5.6%) | 0  (0.0%) | 1  (1.5%) | 2  (2.6%) | 19 (21.1%) | 3  (5.7%) | 0  (0.0%) | 4  (5%) | 0  (0.0%) | 4  (2.4%) |
| UTI-related to catheter | 37  (10.8%) | 6  (6.1%) | 3  (9.7%) | 11  (11.2%) | 8  (15.1%) | 4  (18.1%) | 2  (5.7%) | 52 (8.8%) | 0  (0.0%) | 7  (10.8%) | 1  (1.3%) | 14 (15.5%) | 2  (3.8%) | 5  (17.2%) | 10 (12.2%) | 0  (0.0%) | 13  (7.7%) |
| Post-surgical meningitis | 13  (3.8%) | 2  (2.0%) | 0  (0.0%) | 7  (7.1%) | 1  (1.9%) | 0  (0.0%) | 2  (5.7%) | 18 (3.1%) | 0  (0.0%) | 1  (1.5%) | 1  (1.3%) | 0 (0.0%) | 4  (7.5%) | 2  (6.9%) | 5 (6.1%) | 0  (0.0%) | 5  (3.0%) |
| Surgical site infection | 15  (4.3%) | 3  (3.1%) | 2  (6.5%) | 6  (6.1%) | 0  (0.0%) | 0  (0.0%) | 2  (5.7%) | 28 (4.8%) | 0  (0.0%) | 1  (1.5%) | 6  (7.9%) | 4 (4.4%) | 5  (9.4%) | 1  (3.4%) | 2  (2.4%) | 0  (0.0%) | 9  (5.4%) |
| Intraabdominal infection | 32  (9.3%) | 2  (2.0%) | 0  (0.0%) | 10  (10.2%) | 12  (22.6%) | 6  (27.2%) | 2  (5.7%) | 52 (8.8%) | 0  (0.0%) | 2  (3.1%) | 1  (1.3%) | 20 (22.2%) | 4  (7.5%) | 0  (0.0%) | 9 (11.0%) | 0  (0.0%) | 16  (9.5%) |
| Skin and soft tissue | 15  (4.3%) | 2  (2.0%) | 1  (3.2%) | 6  (6.1%) | 2  (3.8%) | 0  (0.0%) | 2  (5.7%) | 23 (3.9%) | 0  (0.0%) | 7  (10.8%) | 1  (1.3%) | 2 (2.2%) | 2  (3.8%) | 2  (6.9%) | 0 (0.0%) | 0  (0.0%) | 9  (5.4%) |
| Osteoarticular infection | 8  (2.3%) | 0  (0.0%) | 2  (6.5%) | 2  (2.0%) | 0  (0.0%) | 0  (0.0%) | 2  (5.7%) | 18 (3.1%) | 0  (0.0%) | 0  (0.0%) | 2  (2.6%) | 0 (0.0%) | 4  (7.5%) | 1  (3.4%) | 1  (1.2%) | 4  (80.0%) | 6  (3.6%) |
| Other | 8  (2.3%) | 0  (0.0%) | 1  (3.2%) | 3  (3%) | 0  (0.0%) | 1  (4.5%) | 1 (2.9%) | 15 (2.6%) | 1  (5.0%) | 0  (0.0%) | 3  (3.9%) | 2 (2.2%) | 1  (1.9%) | 1  (3.4%) | 2  (2.4%) | 0 (0.0%) | 5  (3.0%) |
| Total number | 344  (100%) | 98  (100%) | 31  (100%) | 98  (100%) | 53  (100%) | 22  (100%) | 35  (100%) | 588 (100%)d | 20  (100%) | 65  (100%) | 76  (100%) | 90  (100%) | 53  (100%) | 29(100%) | 82  (100%) | 5  (100%) | 168 (100%) |

Table S5. Mechanisms of resistance detected among MDRO recovered

| Mechanism of resistance | Number of mechanisms  reported | Number of patients with reported mechanism | Distribution per patient |
| --- | --- | --- | --- |
| MBL | 82 (34.5%) | 75 (35.0%) | 1 in 69 patients (69/75, 92%)  2 in 5 patients (5/75, 7%)  3 in 1 patient (1/75, 1%) |
| KPC | 65 (27.3%) | 58 (27.1%) | 1 in 53 patients (53/58, 91%)  2 in 3 patients (3/58, 5%)  3 in 2 patients (2/58, 3%) |
| ESBL | 63 (26.5%) | 59 (27.6%) | 1 in 57 patients (57/59, 97%)  2 in 3 patients (2/59, 3%) |
| OXA | 26 (10.9%) | 20 (9.3%) | 1 in 14 patients (14/20, 70%)  2 in 6 patients (6/20, 30%) |
| AmpC | 2 (0.8%) | 2 (0.9%) | 2 in 1 patient (100%) |
| Total number | 238 (100%) | 214 (100%) |  |

CPE: carbapenemase-producing *Klebsiella*; MBL: metallo-beta-lactamases; ESBL: extended spectrum β-lactamase producing microorganisms; Oxa: oxacillininases; AmpC: AMPC beta-lactamase.

Data are expressed as numbers (n) and percentages (%).

Table S6. Missing data by patient characteristics

| Variable | Number of patients with missing  data (of 1799 individuals) |
| --- | --- |
| Age | 4 |
| Gender | 0 |
| Risk factors |  |
| Respiratory disease | 4 |
| Obesity | 2 |
| Diabetes | 1 |
| Alcohol use disorder | 5 |
| Smoker | 2 |
| Cardiovascular disease | 2 |
| Chronic liver disease | 4 |
| Chronic renal disease | 1 |
| Immunosuppression | 1 |
| Bone marrow transplantation | 1 |
| Solid organ transplantation | 1 |
| Hematology-oncology diagnosis | 1 |
| Chemotherapy in the previous 6 months | 1 |
| Human immunodeficiency virus status | 1 |
| Any immune deficiency | 0 |
| Hospital admission in the prior 6 months | 0 |
| Use of antibiotics in the prior 6 months | 1 |
| MDRO colonization in the prior 6 months | 1 |
| Days from hospital admission to diagnosis of infection | 81 |
| Days from ICU admission to enrollment | 40 |
| Colonization by MDRO during ICU stay (before the study day) | 1 |
| Location of infection acquisition:  Community/ICU/hospital non-ICU/long-term care | 26 |
| **Data on admission** |  |
| APACHE II score | 16 |
| SOFA score at admission | 5 |
| SOFA score at enrollment | 6 |
| Type of admission  (medical/elective surgery/ emergency surgery) | 1 |
| Trauma admission | 2 |
| Clinical status at enrollment | 20 |

**MANUAL OF OPERATIONS**

**PREVALENCE STUDY ON ANTIMICROBIAL RESISTANCE: COLONIZATION AND INFECTION BY MULTIDRUG-RESISTANT ORGANISMS AND ITS IMPACT ON MORTALITY IN ADULT AND PEDIATRIC CRITICAL CARE IN ARGENTINA**.

**(PREV-AR STUDY)**

1. **SUMMARY OF THE STUDY**

Antimicrobial resistance (AMR) is an urgent global public health threat. During 2019, six pathogens (*E. coli, Staphylococcus aureus, K. pneumoniae, S. pneumoniae, Acinetobacter baumannii*, and *Pseudomonas aeruginosa*) were responsible for 929,000 of 1.27 million AMR-attributable deaths and 3.57 million of 4.95 million AMR-associated deaths worldwide in 2019.(1)

There is a gap in knowledge in the current prevalence of multidrug resistant organisms (MDRO) and its impact on mortality in South America, particularly in critical care patients. Hence, the Argentine Societies of Infectious Diseases (SADI) and Intensive Care Medicine (SATI) will carry out a cross-sectional study aiming to determine the prevalence, risk factors, and associated mortality of infections produced by multidrug resistant organisms (MDRO), and the prevalence of colonization.

**II. INTRODUCTION AND FUNDAMENTALS**

Infection and colonization by multidrug resistant microorganisms (MDRO) represent a great challenge given their association with increased mortality and morbidity (1-4). Comprehensive data on infections produced by MDRO over the world describing risk factors, sites of infection, and particular microorganisms as well as their impact on health outcomes play a pivotal role in increasing awareness among clinicians, patients, caregivers, and stakeholders, particularly in resource-limited settings (2). In addition, this information might aid in developing health policies focused on prevention, diagnosis and treatment of these infections, thus facilitating the adequate allocation of resources (2).

Critically ill patients are particularly vulnerable to colonization and infection produced by MDRO; therefore, knowing its prevalence in the intensive care unit (ICU) is essential to design and implement interventions to improve patient outcomes (3). The European Prevalence of Infection in Intensive Care Study (EPIC III), a 24-hour point-prevalence study conducted at 1150 centers in 88 countries in September 2017, found that 54% of ICU patients had suspected or proven infection. (6)

In recent decades, there has been a rise of antimicrobial resistance (AMR) worldwide and specially in In Latin-America, most healthcare facilities are located in settings with limited resources (2, 4, 5). Most studies describing AMR in this region have focused on colonization or infection without discriminating the impact on ICU patients (5-11). In addition, most data reported belong to the pre-COVID-19 pandemic period, after which the incidence of infections produced by MDROs steeply rose worldwide due to widespread use of antimicrobials and to decreased compliance with infection control practices (12). Therefore, in the aftermath of COVID-19 pandemic, it is essential to update the epidemiology of AMR.

Regarding AMR-associated mortality, a meta-analysis carried out in Latin-American and Caribbean countries found an overall, unadjusted case-fatality rate of 45.0% (4). In studies regarding the burden of AMR carried out in 2019, Latin-America exhibits one of the highest rates of AMR-associated mortality, with an estimate of 569,000-associated deaths, compared to high-income regions (2). These studies, however, were not focused on ICU patients (4, 11, 12). To our knowledge, there is only one study on MDRO-associated ICU mortality in the region, conducted in Brazil, which showed a hospital mortality of 29.5% (13).

Therefore, SADI and SATI will carry out a cross-sectional study aiming to determine the prevalence, risk factors, and associated mortality of infections produced by MDRO, and the prevalence of colonization. We hypothesized that the prevalence of infections caused by MDRO among ICU patients would be higher than 30%, as described in studies of high-prevalence epidemiological situations (14).

.

**III. OBJECTIVES OF THE STUDY**

**Main objectives**

- To estimate ICU mortality in adult patients admitted to ICUs in institutions in Argentina.

**Secondary objectives**

- To estimate the prevalence of infection and colonization by MDRO in adult patients (>18 years) admitted to Intensive Care Units (ICUs) in Argentina.
- To estimate independent determinants for ICU mortality in adult patients admitted to ICUs and prevalence of MDROs
- To estimate the prevalence of Carbapenemase-producing enterobacterales
- To determine the most frequent sites of infections and the microorganisms isolated.
- To determine mortality at day 28.
- To report ICU length of stay

**IV. TYPE OF STUDY DESIGN**

Observational, cross-sectional and descriptive study with longitudinal and prospective follow-up for analysis of ICU mortality to be performed in one day between November 28-30, 2023 at 8.00 a.m. in adult ICUs located in public, social security, and private hospitals in Argentina.

**V. ORGANIZATION OF THE STUDY**

**1. PARTICIPATING UNITS**

All adult ICUs in Argentina that met the inclusion criteria will be invited to participate. The call will be made through SATI and SADI.

**2. GENERAL COORDINATION**

- SATI COMMITTEES: Critical Infectious Diseases Committee.

- FINANCING ENTITY: SADI and SATI.

- DATA SUPERVISION AND SECURITY: Study coordinator hired by SADI - Carina Balasini- Viviana Rodriguez - Damian Aguila.

- PRINCIPAL INVESTIGATORS: Wanda Cornistein - Carina Balasini - Viviana M Rodriguez - Yanina Nuccetelli - Damian Aguila - Alejandra Macchi - Norma Cudmani - Maria Virginia Roca - Graciela Sadino -Soledad E Gonzalez-Elisa Estenssoro.

- LOCAL INVESTIGATORS: Heads of the units and/or infectious diseases or critical care physicians.

- POLICY GUIDELINES: The leaders involved in each hospital's project will be included in the publication(s) derived from this project according to standard journal publication rules. The local leaders will suggest to the principal investigators about any other collaborators who met the criteria and should be considered for inclusion as co-authors (maximum of two co-authors per component per hospital).

Hospitals/local leaders will be able to publish the individual and/or regional results of this research study in a scientific meeting and/or journal by informing in advance and sending a copy to the principal investigators and other regional project leaders for approval. It is not mandatory to include the principal investigators as authors. The following statement should be included in the publication:

"The data reported were obtained as part of a larger study conducted in the region (PREV-AR Project) sponsored by the Argentine Society of Infectious Diseases (SADI) and the Argentine Society of Intensive Care (SATI). The findings and conclusions of this report do not represent the official position of SADI or SATI. The principal investigators may publish the institutional results of this research study in a meeting and/or scientific journal by previously informing and sending a copy to the local leaders of each hospital. For this publication, the principal investigators will be the authors of the manuscript."

**3. RECRUITMENT**

**3.1. ELIGIBILITY**

SADI and SATI will send a Newsletter by email to the members of each society on two occasions, with an interval of 10 days, inviting them to participate in the study, and announced the study on their respective websites.

Each institution interested in participating completed an online form in which general information about the institution will be requested.

Investigators per center: Name and medical specialty (infectious disease specialist or intensivist). Up to 2 investigators per center; if there is more than one ICU in the institution, one investigator per unit surveyed).

The protocol will be sent to each institution for approval.

Once approved by the Ethics Committee of each hospital, hospitals will have to sign a Institutional commitment letter.

**3.2. INCLUSION CRITERIA**

Inclusion criteria for ICUs include:

-Letter of commitment signed by the authorities of the hospital and the critical area to guarantee data collection throughout the study (Appendix 1).

-Hospitals that have a microbiological diagnostic laboratory capable of detecting resistance mechanisms by phenotypic, molecular and/or immunochromatographic methods.

**3.3 EXCLUSION CRITERIA**

Institutions that do not meet the requirements of the inclusion criteria.

**3.4. SAMPLE TYPE AND SAMPLING DESIGN**

Non-probability consecutive sampling

**Target population:** All patients hospitalized in an adult ICUs in hospitals in Argentina during the study period (one day to choose between November 28-30, 2023).

**Accessible population:** All patients hospitalized in adult ICUs in hospitals in Argentina during the study period who agree to participate in the study.

**3.5. INCLUSION OF PARTICIPATING CENTERS AND DATA COLLECTION**

Selection of participating centers: SADI and SATI will send an invitation to participate in the study to all societies’ members via email, announcing three online meetings to explain the project. The invitation will also be published on both societies’ websites and social networks (Facebook, Instagram, Linkedin, and Twitter.) Additionally, the study will be announced in the national meetings of each society.

Hospital coordinators: Each center will have 1 or 2 researchers responsible for the project (preferably 1 intensivist and 1 infectious diseases physician). In the case of centers with several ICUs, 1 additional physician will be admitted from the second ICU onwards.

Each hospital must have a computer with internet connection to download information and upload data.

Hospitals will be registered on a secure website, where local investigators will record main site characteristics in an electronic form.

The following types of institutions will be excluded from this study: ambulatory surgery centers, psychiatric inpatient centers or areas, and neonatology units.

Hospital and Patient data will be entered in an electronic case report form (CRF) using the Research Electronic Data Capture (REDCap) database (Appendix 2). Local researchers will be trained through online meetings on how to fill out the CRF.

.

**4. MEASUREMENTS**

- BASAL MEASUREMENTS: during the day of the study the following data will be completed: patient code; age and sex; type of ICU; date of admission to the hospital and ICU; type of admission; risk factors for MDRO; admission diagnosis; APACHE II and SOFA (at ICU admission), and SOFA on the day of the study; clinical status, use of devices and laboratory on the day of the study; presence of colonization; presence of infection (up to 3) with corresponding site, microorganisms isolated (MDRO or non-MDRO, or no isolation) with date of onset; mechanism of resistance; and antimicrobial use, as empirical, targeted or prophylactic ATBs; old or new ATBs.

-MEASUREMENTS TO BE FOLLOWED OVER TIME AND DURATION OF FOLLOW-UP: The date of discharge from the ICU and outcome, as alive or deceased will be recorded. These variables will be truncated at 60 days from the study day (January 28-30, 2024). 28-day mortality will also be estimated.

-CONTACT PROCEDURES FOR NON-RESPONSIVE PARTICIPANTS:

- Participants who after 7 days of the data collection day have not incorporated any patient into the database will be contacted via cell phone and in case no response an e-mail will be sent. Centers that have not collected data will be excluded.

- After 28 days, an e-mail will be sent reminding participants of the date on which they should check the patient 28-day mortality; and every two weeks until completion of data about discharge or decease.

**5. VARIABLES**

**5.1. PREDICTOR VARIABLES**:

**-HOSPITAL CHARACTERISTICS:**

- Province of the country to which it belongs

-Depending on the management modality, hospitals defined as

- Public: Dependent on the national, provincial or ministries of health.
- Private: For-profit privately funded institutions.
- Of community: Non-profit institutions related to a specific community.
- Social security system: Depend on the management of a social welfare institution

-Adult medical-surgical ICUs: ICU that admits patients ≥ 18 years with medical pathology as well as patients undergoing elective or emergency surgical procedures, prior to or during admission. Includes patients readmitted for a surgical complication. Monovalent units such as burn units, transplant units and polytrauma units can be included in this type of unit.

-Pediatric ICUs: which admit pediatric patients with medical pathology as well as patients undergoing a surgical procedure, prior to or during admission.

-Total number of beds available for use: The total number of beds in the facility that are available for use. Includes both occupied and free beds.

-ICU beds available for use: The total number of beds in the ICU that are available for use. Includes both occupied and free beds.

-Presence of Infection Control Program or Committee: An affirmative answer is given when there is an infection control committee that meets at least three times a year and carries out an institutional infection control program.

-Presence of an antimicrobial stewardship program. An affirmative answer is given when the Institution has a group dedicated to antimicrobial management that includes measurements of defined daily doses and/or treatment days and actions that optimize the use of antimicrobials.

-Methodology used to detect resistance mechanisms must be reported, as

- - Phenotypic methods
  - Molecular methods
  - Immunochromatographic methods
  - More than one Methodology

**PATIENT CHARACTERISTICS**

-Date of entry to the Institution: day-month-year

-Date of admission to the ICU: day-month-year

*-*Age: according to date of birth. In months up to one year of life, then in years.

-Biological sex: female-male

-Comorbid conditions and other risk factors for infection by MDRO:

- Asthma/EPOC: Asthma or Chronic Obstructive Pulmonary Disease. History must be referred to by previous diagnoses or in the medical history.
- Diabetes: The patient will be considered to have diabetes when this is recorded in the history or if blood glucose levels equal to or higher than 145 mg/dl are observed in patients not undergoing fluid therapy that may cause an increase in blood glucose levels. In patients undergoing this type of treatment, levels equal to or higher than 200 mg/dl will be considered.
- Obesity: Body Mass Index ≥30 kg/m^2^
- Alcohol-related problem: chronic disease characterized by uncontrolled and excessive consumption of alcohol that generates strong physical and psychological dependence, manifested through various withdrawal symptoms when it is not possible to ingest it.
- Smoking: chronic addiction characterized by the regular and compulsive consumption of tobacco products, which contain the highly addictive substance known as nicotine that generates physical and psychological dependence.
- Ischemic heart disease: a medical condition characterized by reduced blood flow to the heart muscle due to obstruction or narrowing of the coronary arteries, which can lead to angina pectoris and acute myocardial infarction and its complications.
- Chronic liver disease: a medical condition in which the liver undergoes pathological and abnormal changes that persist for a prolonged period, usually more than 6 months, caused by chronic hepatitis, nonalcoholic hepatic steatosis, liver cirrhosis, chronic alcohol consumption, metabolic and autoimmune diseases.
- Chronic renal insufficiency: The patient will be considered to have chronic renal insufficiency when this is recorded in the history or if creatinine values higher than 1.7 mg/dl are found in blood tests prior to admission.
- Previous infections: episodes of infection in the 3 three months prior to hospitalization, or during the current hospitalization but different from those present on the day of the study.
- Immunosuppressive treatment: patient receiving a treatment that inhibits one or more components of the adaptive or innate immune system.
- Bone marrow transplantation: when the patient underwent a complex procedure in which progenitor cells from a donor are infused into a recipient, with the purpose of restoring marrow function affected by acquired onco-hematological and non-oncological diseases.
- Solid organ transplant: When the patient is a carrier of a solid organ transplant prior to the current admission.
- Onco-hematological neoplasm: Patients diagnosed with hematologic malignant neoplasm in the course of the last 5 years.
- Chemotherapy (last 6 months): Patient who in the last 6 months has received chemotherapy medication.
- HIV: patient with acquired immunodeficiency virus infection.
- Previous admission to a hospital in the last 6 months
- Previous Colonization by MDRO: patient with positive anal swab for Colonization by ERC, PAE DT, CRAB or EVR; or positive nasal swab for Colonization by SAU, SAMR, VISA or VRSA , in the last 6 months previous to the study
- Antibiotic use in the last 6 months: patient who has received antibiotics in the 6 months prior to the current episode.

-Admission diagnosis: This is a drop-down list box, so that when clicking on the right arrow all the possible diagnoses to choose will appear. There is the option to answer other diagnosis, or unknown (at the end of the list).

- Cardiocirculatory: Myocardial infarction, Stable angina, Unstable angina, Complicated acute myocardial infarction, Arrhythmias, Congestive heart failure, Acute cardiogenic pulmonary edema, Cardiorespiratory arrest, Hypertensive crisis, Hypovolemic shock, Cardiogenic shock without infarction, Septic shock, Postoperative cardiac surgery, Sepsis, Polytrauma without cranial trauma, Anaphylactic shock, Others (pericarditis, etc., etc.), Endocardial shock..., etc.), Endocarditis, Vascular surgery postoperative control, Congenital heart disease, Pulmonary hypertension.
- Respiratory: Chronic respiratory insufficiency Chronic respiratory insufficiency exacerbation of COPD, Acute respiratory insuf. and ARDS, Asthma, post-anesthesia ventilatory failure, pulmonary thromboembolism, neoplasms, operated or not, pneumonia or bronchopneumonia, postoperative chest, respiratory failure of other causes, thoracic trauma, others (laryngectomies, etc.), bronchodysplasia, pulmonary hypoplasia, diaphragmatic hernia, bronchiolitis, congenital tracheal stenosis, acquired tracheal stenosis.
- Gastrointestinal: Chronic decompensated liver disease, Pancreatitis, Peritonitis, Digestive fistulas, Upper gastrointestinal bleeding, Hepatitis, Post-operative esophageal neoplasms, postoperative intestinal resection, Other post-operative, Abdominal trauma, Other digestive, Esophageal atresia, Bile duct atresia-stenosis, Duodenal atresia-stenosis, Enterocolitis.
- Central nervous system, Cerebral vascular accident, Non-toxic structural coma, Cranioencephalic trauma (TEC), TEC and other associated traumas, Neurological postoperative, Pharmacological intoxications, Tetanus, Guillain-Barré and others, Seizures, Meningitis or encephalitis, Other central nervous system, Overdose, Congenital encephalopathy, Acquired encephalopathy, Meningitis secondary to ventricular devices, Meningitis secondary to ventricular devices.
- Renal: acute renal failure, acute chronic renal failure, urological postoperative, Other renal
- Metabolic: diabetic decompensation, dehydration, electrolyte alterations, other (includes endocrinopathies), metabolopathies
- Hematological: Intravascular coagulation, other hematologic, hiv/aids, congenital anemia, primary immunodeficiency.
- Traumatological: spinal cord injury, facial trauma, surgery in polytraumatized patients.
- Transplantation: Lung transplantation, Kidney transplantation, Liver transplantation, Heart transplantation, Hematopoietic organ transplantation, Other transplantations
- Burned
- After invasive procedures: ERCP (Endoscopic Retrograde Cholangiopancreatography), other.
- Other diagnosis: Postoperative management of maxillofacial surgery, cellulitis / necrotizing fasciitis, other or unknown diagnosis

-Severity of disease at admission:

It will be evaluated by means of:

- APACHE II: Acute Physiology and Chronic Health disease Classification System II is a disease severity classification system; the minimum score is 0 and the maximum 71.
- SOFA SCORE AT ICU ADMISSION: The Sequential Organ Failure Assessment (SOFA) Score evaluates multiple organ dysfunction and failure. Each organ alteration is scored from 0 to 4. The score is the sum of all the isolated organ assessments. A score other than 0 and less than 3 is considered as organ dysfunction, while higher scores indicate organ failure.
- SOFA SCORE on the day of the study.

The SOFA score ranges from 0 to 24.

-Type of admission

- - Medical: No surgery prior to admission. Patients admitted to the ICU after a minor surgical procedure (permanent pacemaker, ERCP, etc.) that generally do not require deep anesthesia are also considered medical patients. Patients requiring intubation for catheterization of any location and purpose (endovascular prosthesis, embolization, etc.) are also considered medical patients.
  - Elective surgery: When the surgery was scheduled at least 24 hours in advance in the 7 days prior to admission to the ICU.
  - Emergency surgery: It is understood as urgent when the surgery was performed within the first 24 hours of its indication. It includes patients who require urgent surgery and are admitted to the ICU for pre-operative stabilization and/or postoperative control.

Apart from the 3 above mentioned categories, patients will be classified according to admission for trauma or not

- - Trauma: When admission to the ICU was the consequence of open or closed trauma in a patient with or without surgical intervention.

- Clinical status on the day of the study, according to SEPSIS-3 definitions (15)

- No infection
- Infected but no sepsis: No increase in SOFA
- Sepsis: Infection with new organ failure, diagnosed with an increase in SOFA ≥ 2 points.

In this subgroup,

- Septic shock: requires vasopressors to maintain MAP≥65 mmHg after adequate resuscitation with fluids + lactate >2 mmol/L.

- Infection on the day of the study: patients will be further grouped according to the presence or not of any infection on the day of the study. Infections will be considered as definite (microbiologically confirmed), and probable or possible, according to the International Sepsis Forum definitions (ISF). (16). In the cases where culture results were pending, categorisation was reviewed with the main investigators when the results were made available.

Definitions of the ISF:

- **Severe community-acquired pneumonia:** Presence of new-onset infiltrates on chest X-ray, plus history and physical examination compatible with the diagnosis of pneumonia (cough and expectoration, tachypnea/dyspnea, chest pain, crackling rales), acquired prior to hospital admission without having been admitted in the previous 14 days. Severity criteria:

Respiratory rate > 30/min

O_2_ saturation <93% (ambient air)

Requirement for mechanical respiratory support

Increased infiltrates >50% in 24 to 48 hours

Alteration of consciousness - Impairment of sensorium

Hemodynamic instability

CURB-65 ≥2 points

UTI requirement

Multiorgan failure

- **Hospital (HAP) or nosocomial pneumonia**: consists in a lower respiratory infection that was not present at the time of hospital admission and that develops 2 or more days after hospitalization.

Pneumonia is defined as a new lung infiltrate or progression of a previous infiltrate plus clinical evidence that the infiltrate is of an infectious origin, which include the new onset of fever, purulent sputum, leukocytosis, and decline in oxygenation. It maybe suspected when it meets the following criteria:

- - - - - Progressive or new pulmonary infiltrate on chest x-ray persistent for more than 48 hours AND
        - Tracheal aspirate (TA) purulent AND
        - Fever > 38° AND
        - Leukocytosis (WBC>12000) or leukopenia (WBC<4000)
- **Ventilator-associated pneumonia:** Lower respiratory infection that develops after 4 or more days of the initiation of invasive mechanical ventilation.

Pneumonia is defined as a new lung infiltrates or progression of previous infiltrate plus clinical evidence that the infiltrate is of an infectious origin, which include the new onset of fever, purulent sputum, leukocytosis, and decline in oxygenation. It maybe suspected when it meets the following criteria:

- - - - - Progressive or new pulmonary infiltrate on chest x-ray persistent more than 48 hours AND
        - Tracheal aspirate (TA) purulent AND
        - Fever > 38° AND
        - Leukocytosis (WBC>12000) or leukopenia (WBC<4000)

Each type of pneumonia will be further characterized as:

- **Definite or microbiologically confirmed pneumonia:** The patient must have a new or progressive radiographic infiltrate, along with a high clinical suspicion of pneumonia (or a CPIS >6, using a Gram stain of a lower respiratory tract sample) plus a definite cause established by the recovery of a probable etiologic agent from a) an uncontaminated specimen (blood, pleural fluid, transtracheal aspirate, or transthoracic aspirate); b) the recovery from respiratory secretions of a likely pathogen that does not colonize the upper airways (e.g., *Mycobacterium tuberculosis*, *Legionella* species, influenza virus, or *Pneumocystis jiroveci (carinii*); c) recovery of a likely/possible respiratory pathogen in high concentrations using quantitative cultures of a lower respiratory tract sample (endotracheal aspirate, BAL, or protected specimen brush); or d) positive serology.
- **Probable pneumonia:** The patient must have a new or progressive radiographic infiltrate along with a high clinical suspicion of pneumonia (or a CPIS of _6, using a Gram stain of a lower respiratory tract sample) plus detection (by staining or culture) of a likely pulmonary pathogen in respiratory secretions (expectorated sputum, endotracheal or bronchoscopic aspirate, or quantitatively cultured bronchoscopic BAL fluid or brush catheter specimen), but in concentrations below the diagnostic threshold, or the presence of a negative lower respiratory tract culture if collected within 72 hrs after starting a new antibiotic regimen.
- **Possible pneumonia**: Abnormal chest radiograph of uncertain cause, in a patient with a low or moderate clinical suspicion of pneumonia, but with microbiological or serological evidence of definite or probable pneumonia (as defined above).
- **Urinary tract infection acquired in the community** (non catheterized patients)

This infection must be acquired prior to hospital admission, with no history of hospitalization within the previous 14 days.

- - - - Lower urinary tract infection is usually not considered as a possible source of severe sepsis/septic shock, but if required the conventional microbiological definition of ≥10^5^ cfu/ mL can be used.
      - Upper urinary tract infection (kidney, ureter, or tissue surrounding the retroperitoneal or perinephric space).

Must meet one of the following criteria:

- Organism isolated from culture of blood or any other fluid (other than urine) or tissue from the affected site
- An abscess or other evidence of infection seen on direct examination, during surgery, or by histopathologic examination

Or two of the following:

- Fever (>38°C); urgency; localized pain or tenderness at involved site; and any one of the following: microscopic examination (urinalysis or Gram stain) showing pyuria or ≥10^5^ cfu/mL; purulent drainage from the affected site; pyuria; hematuria; organism isolated from urine culture; positiveGram stain; radiographic evidence of infection (e.g., ultrasound, computed tomography, magnetic resonance imaging, radiolabeled scan).cfu, colony-forming units.
- **Urinary tract infection related to a urinary catheter**

Patients with an indwelling urinary catheter placed for > 2 days at the event date, or that has been removed within the past 6 days.

- Lower urinary tract infection: presence of suggestive signs and symptoms including fever (>38°C), urgency, frequency, dysuria, pyuria, hematuria, positive Gram stain, pus, suggestive imaging

and pyuria (≥10 white blood cells/L or ≥3 white blood cells/high-power field of unspun urine)

**or** organisms seen on Gram stain of unspun urine

**or** frank pus expressed around the urinary catheter

**or** >10^3^ cfu/mL **or** if the patient can report symptoms, modified CDC criteria have to be met

- Upper urinary tract infection (kidney, ureter, bladder, urethra, or tissue surrounding the retroperitoneal or perinephric space)

Must meet **one** of the following criteria: Organism isolated from blood culture of fluid (other than urine) or tissue from the affected site; an abscess or other evidence of infection seen on direct examination, during surgery, or by histopathologic examination

or **two** of the following:

Fever (>38°C), urgency, localized pain or tenderness at involved site, and any of the following: purulent drainage from the affected site, pyuria, hematuria, organism isolated from culture, positive Gram stain, radiographic evidence of infection (e.g., ultrasound, computed tomography, magnetic resonance imaging, radiolabeled scan.

- Modified CDC criteria:

One of the following: fever (>38°C), urgency, frequency, dysuria or suprapubic tenderness, **and** a urine culture ≥10^5^ cfu/mL with no more than two species of organisms

**or**

2 of the following: fever (>38°C), urgency, frequency, dysuria or suprapubic tenderness,

**and any** of the following:

-positive dipstick for leukocyte esterase and/or nitrate

-pyuria (≥10 white blood cells/_L or ≥3 white blood cells/high-power field of unspun urine)

-organisms seen on Gram stain of unspun urine

-2 urine cultures with repeated isolation of the same uropathogen with ≥10^2^ cfu/mL in nonvoided specimen

-2 urine cultures with ≤10^5^ cfu/mL of single uropathogens in a patient being treated with appropriate antimicrobial therapy

- **Definite catheter-related blood infection** **with bacteriologic confirmation** is defined as at least one peripheral positive blood culture and one of the following, in a patient with a central line:

-A positive semiquantitative (≥15 colony-forming units [cfu]/catheter segment) or quantitative (≥10^3^ cfu/catheter segment) catheter tip culture (i.e., catheter Colonization), whereby the same microorganism (species and antibiogram) is isolated from the catheter segment and peripheral blood

-A positive hub or exit site culture growing the same microorganism as peripheral blood

**or**

-Positive paired central and peripheral blood cultures growing the same organism, where the central blood culture is positive ≥2 hrs earlier than the peripheral blood culture or has five times the growth of the peripheral blood culture.

AND the organism(s) identified in blood is not related to an infection at another site.

In the case of skin commensals, the patient has at least 2 positive blood cultures with the same microorganisms. Patients of any age have a recognized bacterial or fungal pathogen, not included on the NHSN common commensal list.

- **Primary bacteremia:** Patient must meet the following two criteria:

Patient has a recognized pathogen (defined as a microorganism not usually regarded as a common skin contaminant, i.e., diphtheroids, *Bacillus* species, *Propionibacterium* species, coagulase-negative staphylococci, or micrococci) cultured from one or more blood cultures

**or**

A common skin contaminant (e.g., diphtheroids, *Bacillus* species, *Propionibacterium* species, coagulase-negative staphylococci, or micrococci) cultured from two or more blood cultures drawn on separate occasions (including one drawn by venipuncture)

**and**

The organism cultured from blood is not related to an infection at another site, including intravascular-access devices.

- **Intraabdominal infections** They are broadly defined as presence of peritoneal inflammation in response to microorganisms, resulting in purulence in the peritoneal cavity. They are classified as uncomplicated or complicated based on the extent of infection.

**Uncomplicated intraabdominal infections**involve intramural inflammation of the gastrointestinal tract without anatomic disruption

**Complicated intraabdominal infections**extend beyond the source organ into the peritoneal space. They cause peritoneal inflammation, and are associated with localized or diffuse peritonitis. Localized peritonitis often manifests as an abscess with tissue debris, bacteria, neutrophils, macrophages, and exudative fluid contained in a fibrous capsule. Diffuse peritonitis is categorized as primary, secondary or tertiary peritonitis.

- **Primary peritonitis** (also referred to as spontaneous bacterial peritonitis) is defined as a microbial infection of the peritoneal fluid in the absence of a gastrointestinal perforation, abscess, or other localized infection within the gastrointestinal tract
- *Microbiologically confirmed*: the presence of a clinically compatible presentation of primary peritonitis with the isolation of microbial pathogens (in peritoneal fluid or blood) along with evidence of acute inflammatory reaction within the peritoneal fluid (i.e.>500 leukocytes/mL) with a neutrophilic predominance, an ascitic fluid pH <7.35 (arterial to ascitic pH difference of <0.1), or a lactate concentration >2.5 mg/L
- *Probable*: Clinically appropriate setting with evidence of an inflammatory ascitic fluid (>500 leukocytes/mL with a neutrophil predominance) in the presence of a positive Gram stain but negative peritoneal fluid cultures or in the presence of a positive blood culture for a pathologic organism with inflammatory cells in ascitic fluid
- *Possible*: A compatible clinical illness with an inflammatory peritoneal fluid (>500 leukocytes/mL) in the absence of a positive culture (in peritoneal fluid or blood) or Gram stain
- **Secondary peritonitis** is a microbial infection of the peritoneal space following perforation, abscess formation, ischemic necrosis, or penetrating injury of the intra-abdominal contents
- *Microbiologically confirmed*: Isolation of one or more microbial pathogens found in the peritoneum or the blood >24 hrs after a gastrointestinal perforation of the stomach, esophagus or duodenum, or any perforation of the small bowel distal to the ligament of Treitz. Spillage of luminal contents during an operative procedure is not sufficient evidence of perforation that allows for definitive diagnosis of peritonitis. Furthermore, apenetrating abdominal wound or documented perforation that is surgically repaired within 12 hrs of its occurrence is not sufficient evidence to support diagnosis of secondary bacterial peritonitis.
- *Probable*: Compatible clinical illness associated with documented evidence of perforation (free air in the abdomen on radiographic studies or surgical confirmation of peritoneal inflammation following luminal perforation in the absence of microbiological confirmed peritonitis). A Gram stain in the absence of a positive culture from the peritoneum would be considered probable secondary bacterial peritonitis.
- *Possible*: Upper gastrointestinal perforation or penetrating abdominal trauma that is surgically repaired without further evidence of microbiologic confirmation or clinical signs or symptoms supportive of a diagnosis of bacterial or fungal peritonitis.

A finding of an inflammatory peritoneal fluid in the presence of a documented but localized intra-abdominal abscess in the absence of culture confirmation would also be considered possible secondary bacterial peritonitis.

- **Tertiary peritonitis** is defined as persistent intra-abdominal inflammation and clinical signs of peritoneal irritation following secondary peritonitis from nosocomial pathogens.
- *Microbiologically confirmed*: Isolation of one or more nosocomial pathogens from peritoneal fluid or blood in an appropriate clinical situation (>48 hrs after treatment for primary or secondary peritonitis).
- *Probable*: Compatible clinical illness with documented secondary peritonitis with persistent peritoneal inflammation (>500 leukocytes/mL peritoneal fluid) in the absence of microbiologically confirmed microbial persistence in the peritoneal space.
- *Possible*: Compatible clinical illness with persistent signs of systemic inflammation but without clear documented evidence of persistent inflammation within the peritoneal space following secondary bacterial peritonitis.
- **Intra-abdominal abscess**
- *Microbiologically confirmed*: Clinical, radiographic, and direct surgical confirmation of an inflammatory collection within the peritoneal space or surrounding structures with isolation of one or multiple microbial pathogens from the fluid collection. Microbiologic confirmation will require specimen collection from percutaneous aspirations under sterile technique or direct surgical observation with acquisition of culture material directly from the abscess cavity or the blood.
- *Probable*: The presence of an abnormal collection of fluid in the intra-abdominal contents or surrounding structures with evidence of inflammatory
- cells and/or positive Gram stain but with negative cultures from that fluid accumulation or blood.
- *Possible*: Clinical or radiographic evidence of an abnormal fluid accumulation within the abdominal contents or surrounding structures but without microbiologic or surgical confirmation.
- ***C. difficile* diarrhea**

Diarrhea or evidence of megacolon or severe ileus with positive stool laboratory test for *C. difficile* and/or evidence of pseudomembranes by endoscopy or histopathology.

- **Surgical site infection (SSI)**

Is defined as an infection that arises within 30 days of an operative procedure and at the site of surgical intervention. Symptoms and signs suggestive of a surgical site infection include wound erythema and blanching, tenderness, pain, purulent discharge, fever (temperature >38.0°C), and leukocytosis. A superficial surgical site infection involves the skin or subcutaneous tissues alone, whereas a deep surgical site infection involves the fascia or muscle layers, and an organ space surgical site infection involves the deeper anatomic areas opened during the surgical procedure.

- **Skin/soft tissue infections**
- *Cellulitis* is defined as an acute spreading infection of the skin and underlying soft tissue suggested by the presence of a rapidly expanding erythema, local tenderness, pain, swelling, lymphangitis, and lymphadenopathy, which is frequently accompanied by systemic signs and symptoms including malaise, fever (temperature >38.0°C), and chills.
- *Necrotizing cellulitis and fasciitis* are defined as acute, rapidly progressing, and life-threatening destructive (i.e., necrotizing) infections of the subcutaneous tissues dissecting along tissue planes. Although these two clinical entities exhibit some distinctive clinical and microbial characteristics, they share common features. The symptoms and signs suggestive of necrotizing cellulitis or fasciitis intense local pain (a cardinal feature), exquisite tenderness, erythema (initially discrete but evolving to red-purple and then blue-gray cutaneous lesions often with hemorrhagic bullae), swelling, edema, crepitations (in the case of necrotizing cellulitis), and extensive tissue necrosis, which are associated with prominent systemic toxicity (toxic shock syndrome, severe sepsis, or septic shock).
- **Cardiovascular infection**: Signs and symptoms compatible with infections of the cardiovascular system such as native or prosthetic valve endocarditis; infection of pacemakers or devices; mediastinitis.
- **Osteoarticular infection:** presence of clinical pictures compatible with infections of the osteoarticular system: septic arthritis (including prosthetic infection) and osteomyelitis.
- **Nosocomial meningitis**

Criterion 1: Positive culture of cerebrospinal fluid (CSF)

Criterion 2: at least one of the following: clinical findings with no other known cause: headache, fever (>38^o^C), meningeal signs, cranial nerve signs or irritability. Plus one of the following: a). increased leukocytes, protein and/or decreased glucose in CSF. b). positive Gram stain in CSF. c). Positive blood culture. d). Positive antigen test in CSF, blood or urine. e). Diagnosis of high IgM titers or IgG serologic pair.

Criterion 3: Patients aged <1 year, with at least two of the following signs or symptoms, with no other known cause: fever (>38oC rectally), hypothermia (<37oC rectally), apnea, meningeal signs, bradycardia, cranial nerve signs or irritability. Plus one of the following: a) increased leukocytes, protein and/or decreased glucose in CSF. b) positive Gram stain in CSF. c) positive blood culture. d) positive antigen test in CSF, blood or urine. e) diagnosis of high titers of IgM or IgG serologic pair.

- **Microorganismos isolated:** MDRO of interest are defined according to the WHO list of priority pathogens for research and development of new antibiotics considering their impact on the outcomes of hospitalized patients**. (17)**

- Carbapenem-resistant *Acinetobacter baumannii* (CRAB)

*- Difficult-to-treat Pseudomonas aeruginosa:* resistant to first-line drugs simultaneously: antipseudomonal cephalosporins, aztreonam, piperacillin tazobactam, imipenem, meropenem and fluoroquinolones (DT-PAE).

- carbapenemase-producing enterobacterales (CPE)

- Extended-spectrum beta-lactamase (ESBL)-producing *Enterobacteriaceae* (ESBL)

*-Enterococcus faecium*, vancomycin-resistant (EVR)

*- Staphylococcus aureus* (SAU), methicillin-resistant (MRSA).

Fungi, parasites and viruses will be excluded.

-**Colonization** is defined as a positive rectal sample in patients without signs and symptoms of infection. obtained in the ICU or previously to ICU admission. The date of colonization will be recorded. The frequency of surveillance is defined by each hospital, as weekly, at ICU admission and/or previous to surgery.

-**Mechanisms of resistance of MDRO recorded**: If available, diagnostic methods will be performed to discriminate the mechanism of antibacterial resistance for each microorganism: carbapenemase-producing enterobacterales (CPE*)*, metallo-beta-lactamases (MBL), extended spectrum β-lactamase (ESBL) producing organisms, AMPC beta-lactamases, and oxacillinases (OXA).

-**Non-MDRO microorganisms** recorded: include infections by *S.pneumoniae, S.pyogenes, S.aureus, E.Coli,,* coagulase-negative *Staphylococcus*, *Proteus* sp., KES (*Klebsiella-Enterobacter-Serratia-Citrobacter* (KESC) group, *C. difficile*, and others.

**-Antibiotic treatment.**

The prescribed antimicrobial treatment is classified as:

- Empiric or targeted: initial treatment due to suspicion of infection without rescue of the causal germ. Empiric antimicrobial therapy: defined as the initial antibiotic regimen selected in the absence of definitive microbiological pathogen identification and susceptibility testing. It is usually based on the probable source, patient location at the time of acquisition, patient characteristics and risk factors and local epidemiological data in the unit. Targeted therapy: targeted or definitive therapy is the antibiotic regimen selected after pathogen identification and susceptibility testing is completed. (18)
- Adequate or inadequate. Adequate: defined as microbiologically effective antimicrobial treatment against the causative pathogen. This process usually implies narrowing of the spectrum of antimicrobial coverage on the basis of culture results. (19)
- Receiving novel or traditional antibiotics (20)

Traditional: Infections treated with beta-lactams +/- beta-lactamase inhibitors (sulbactam, tazobactam), quinolones, aminoglycosides, fosfomycin, colistin, clindamycin, metronidazole directed to isolated microorganisms.

Novel antibiotics: treatment directed to the causative microorganism: a. KPC and OXA treated with ceftazidime-avibactam; MBL treated with avibactam+aztreonam, PAEDT with ceftolozane-tazobactam or imipenem relebactam.

- Receiving Access, Watch and Reserve antibiotic groups according to the AWaRe WHO classification.(21). See list on Appendix 3.

**5.2. OUTCOME VARIABLES**

**MAIN OUTCOME VARIABLE**

- Prevalence of infection and colonization by MDRO in adult patients (>18 years) admitted to Intensive Care Units (ICUs) in Argentina

**SECONDARY OUTCOME VARIABLES**

- ICU mortality in patients admitted to ICUs in institutions in Argentina and its independent determinants.
- Characteristics and mortality of patients with infections by MDRO vs. no MDRO. Independent risk factors for infections by MDRO.
- Frequency of the different sites of infections and of the microorganisms isolated
- Frequency of mechanisms of resistance of Carbapenemase-producing enterobacterales, a subgroup of MDRO.
- Characteristics and mortality of patients with infections in general vs. without infections.

**VI. QUALITY CONTROL**

1. GENERAL AND RESPONSIBILITIES

1.1 The principal investigators of the study will be responsible for the preparation of the protocol, forms and operations manual. They will also be responsible for calling the centers, controlling compliance with the requirements for participation, receiving and evaluating the letters of commitment from the centers, as well as monitoring the approvals of the Ethics Committees.

They will be responsible for the confidentiality of the data, return of results and preparation and dissemination of the final report, as well as for any publications that may arise from this study.

Hospital coordinators shall be responsible for the following activities:

- Fill in the form with the institution's data (Appendix).
- Send the Participation Agreement (Appendix) duly signed by the hospital investigators, heads of the ICUs and the Hospital Director.
- Send the Ethics Committee/Institutional Teaching Committee Approval as appropriate (Appendix) duly signed.
- Carry out data collection and upload the required data to the system.
- In case the institutional Ethics/ Committee requests informed consent, request it for each patient.
- Communicate to the principal investigators any eventuality related to the study.
- Follow up each patient and complete mortality at 28 days after the prevalence cutoff.

2. TRAINING IN THE PROCEDURES TO BE PERFORMED

Two training meetings will be scheduled in October for understanding the forms and including their contents in RedCap.

3. EXTERNAL REVIEW AND VISITS TO THE CENTRE (If applicable)

No visits to the center are required. Completion of the forms will be checked weekly until the end of the study.

4. EQUIPMENT MAINTENANCE (If applicable)

**VII. DATA MANAGEMENT**

-Data collection

Each participating institution will complete an online form requesting general information about the institution.

The data will be loaded by means of REDCap software (Research Electronic Data Capture).

A day will be defined between November 28 and 30 on which each institution will collect data. All data will be collected on a case report form (CRF) designed for this purpose (see Annex 3).

-Data input

REDCap software will be used to create the database. Each institution will be provided with a username and password to enter the data collected.

-Editing, storage and backup

REDCap software allows data to be stored on secure servers. A backup is performed every night.

-Missing data will be presented.

-Confidentiality of data

Each institution will be assigned a code to be used during data collection. Likewise, the patient will be registered in the CRF under a code composed of the first letter of his/her first name, followed by the first letter of his/her last name and finally the date of birth. Each CRF is for EXCLUSIVE USE within the hospital, in no case may it be removed from the hospital, reproduced or disseminated by any means. The access to the REDCap platform to upload the data will be through the user assigned to the participants. Participants will not share their login credentials with other study personnel working on the project. Uploading data into REDCap will only allow you to complete the ID number. Both during the study and after completion, each site will be able to independently access its own data through the web platform. At all times the confidentiality of each center will be preserved through an automatic coding system. At no time will the reported data be directly linked to the center of origin.

**VIII. DATA ANALYSIS PLAN**

- Form of data presentation

A unified REDcap database will be created with the data of all patients included in the study on the prevalence day.

-Sample size: Given that this is an observational study and there is no risk to patients, we sought to include as many patients as possible, with no predefined sample size.

Prevalence will be calculated as the number of infections produced by MDRO/number of patients at the ICU on the day of the study.

Variables will be reported as absolute numbers and percentages, means and standard deviations, or medians and percentiles [0.25-0.75]. Differences in the variables recorded between patients with MDRO and non-MDRO infections, between survivors and non-survivors, and between patients with and without infection, will be analyzed with χ² tests or Fisher's exact test, with the t-test, or with the Wilcoxon rank sum test, as appropriate to the nature and distribution of each variable. A p value of <0.05, two-tailed will be considered statistically significant. The Bonferroni correction will be used to adjust for multiple comparisons when necessary.

To estimate associations of patient characteristics, ICU organizational factors, and hospitals, with infection by MDRO and also with ICU mortality, we will use mixed-effects models with the structure of a patient (level 1) admitted to a hospital (level 2). Patients will be thus nested within hospitals. Only variables that have a *P* value <.20 in the bivariable analysis will be introduced in the final model. The dependency between patients treated at a hospital will be captured through the use of the random intercepts. The results of the fixed-effects (measures of association) measures will be expressed as odds ratios (ORs) and 95% CIs. Random-effects (measures of variation) measured included the variance, its SE, and the median OR. The statistical significance of covariates will be calculated using the likelihood ratio test.

Data will be analyzed with Stata 14.0 (StataCorp LP, College Station, TX, USA).

**IX. ETHICAL ISSUES**

Each local institutional review board has to approve the study and establish the requirement for informed consent. Letters asking for exception of informed consent will be provided at the request of local investigators (Appendix 4).

**X. REFERENCES**

1. [Gaudet A, Kreitmann L, Nseir S. ICU-Acquired Colonization and Infection Related to Multidrug-Resistant Bacteria in COVID-19 Patients: A Narrative Review. Antibiotics. 2023; 12(9):1464. https://doi.org/10.3390/antibiotics12091464](about:blank)
2. [Antimicrobial Resistance Collaborators. (2023). The burden of antimicrobial resistance in the Americas in 2019: a cross-country systematic analysis. *Lancet Regional Health. Americas*, *25*, 100561.](http://paperpile.com/b/eGqgWP/873r)
3. [Riley, M. M. (2021). *Infection Challenges in the Critical Care Unit, An Issue of Critical Care Nursing Clinics of North America*. Elsevier Health Sciences.](http://paperpile.com/b/eGqgWP/dR11)
4. Ciapponi A, Bardach A, Sandoval MM, Palermo MC, Navarro E, Espinal C, Quirós R. Systematic Review and Meta-analysis of Deaths Attributable to Antimicrobial Resistance, Latin America. Emerg Infect Dis. 2023 Nov;29(11):2335-2344. doi: 10.3201/eid2911.230753. PMID: 37877573; PMCID: PMC10617342.
5. [Staneloni, M. I., Alonso, L., Ilari, S., Herrera, M. P., López, C., & Colque, Á. (2022). [Program for the prevention of Carbapenemase-Producing Enterobacteria in critical units in Argentina during the COVID-19 pandemic]. *Medicina*, *82*(5), 722–731.](http://paperpile.com/b/eGqgWP/bMr0)
6. [Vincent, J.-L., Sakr, Y., Singer, M., Martin-Loeches, I., Machado, F. R., Marshall, J. C., Finfer, S., Pelosi, P., Brazzi, L., Aditianingsih, D., Timsit, J.-F., Du, B., Wittebole, X., Máca, J., Kannan, S., Gorordo-Delsol, L. A., De Waele, J. J., Mehta, Y., Bonten, M. J. M.,  EPIC III Investigators. (2020). Prevalence and Outcomes of Infection Among Patients in Intensive Care Units in 2017. *JAMA: The Journal of the American Medical Association*, *323*(15), 1478–1487.](http://paperpile.com/b/eGqgWP/nQdZ)
7. [Brooke M Ramay, Carmen Castillo, Laura Grajeda, Lucas F Santos, Juan Carlos Romero, Maria Renee Lopez, Andrea Gomez, Mark Caudell, Rachel M Smith, Ashley Styczynski, Carolyn T A Herzig, Susan Bollinger, Mariangeli Freitas Ning, Jennifer Horton, Sylvia Omulo, Guy H Palmer, Celia Cordon-Rosales, Douglas R Call, Colonization With Antibiotic-Resistant Bacteria in a Hospital and Associated Communities in Guatemala: An Antibiotic Resistance in Communities and Hospitals (ARCH) Study, Clinical Infectious Diseases, Volume 77, Issue Supplement_1, 1 July 2023, Pages S82–S88,](about:blank) <https://doi.org/10.1093/cid/ciad222>
8. Rafael Araos, Rachel M Smith, Ashley Styczynski, Felipe Sánchez, Johanna Acevedo, Lea Maureira, Catalina Paredes, Maite González, Lina Rivas, Maria Spencer-Sandino, Anne Peters, Ayesha Khan, Dino Sepulveda, Loreto Rojas Wettig, María Luisa Rioseco, Pedro Usedo, Pamela Rojas Soto, Laura Andrea Huidobro, Catterina Ferreccio, Benjamin J Park, Eduardo Undurraga, Erika M C D’Agata, Alejandro Jara, Jose M Munita, High Burden of Intestinal Colonization With Antimicrobial-Resistant Bacteria in Chile: An Antibiotic Resistance in Communities and Hospitals (ARCH) Study, *Clinical Infectious Diseases*, Volume 77, Issue Supplement_1, 1 July 2023, Pages S75–S81, <https://doi.org/10.1093/cid/ciad283>
9. Vargas JM, Moreno Mochi MP, López CG, Alarcón JA, Acosta N, Soria K, Nuñez JM, Villafañe S, Ramacciotti J, Del Campo R, Jure MA. Impacto de un programa de vigilancia activa y medidas de control de infecciones sobre la incidencia de bacilos gram negativos resistentes a carbapenems en una unidad de cuidados intensivos [Impact of an active surveillance program and infection control measures on the incidence of carbapenem-resistant gram-negative bacilli in an intensive care unit]. Rev Argent Microbiol. 2022 Apr-Jun;54(2):134-142. Spanish. doi: 10.1016/j.ram.2021.03.003. Epub 2021 Jun 2. PMID: 34088536.
10. Echavarría GL, Guevara Nuñez D, Bertona E, De Paulis AN, Predari SC, Benchetrit G. Colonización por Klebsiella pneumoniae productora de carbapenemasa tipo KPC en un Hospital Universitario [KPC-producing Klebsiella pneumoniae Colonization at a University Hospital]. Medicina (B Aires). 2017;77(2):105-110. Spanish. PMID: 28463215.
11. Ghosh S, Bornman C, Zafer MM. Antimicrobial resistance threats in the emerging COVID-19 pandemic: where do we stand? J Infect Public Health. 2021;14:555–60.
12. Antimicrobial Resistance Collaborators. Global burden of bacterial antimicrobial resistance in 2019: a systematic analysis. Lancet. 2022 Feb 12;399(10325):629-655. doi: 10.1016/S0140-6736(21)02724-0. Epub 2022 Jan 19. Erratum in: Lancet. 2022 Oct 1;400(10358):1102. PMID: 35065702; PMCID: PMC8841637.
13. Martins APS, da Mata CPSM, Dos Santos UR, de Araújo CA, Leite EMM, de Carvalho LD, Vidigal PG, Vieira CD, Dos Santos-Key SG. Association between multidrug-resistant bacteria and outcomes in intensive care unit patients: a non-interventional study. Front Public Health. 2024 Jan 8;11:1297350. doi: 10.3389/fpubh.2023.1297350. PMID: 38259738; PMCID: PMC10801015.
14. Serra-Burriel M, Campillo-Artero C, Agodi A, Barchitta M, López-Casasnovas G. Association between antibiotic resistance in intensive care unit (ICU)-acquired infections and excess resource utilization: Evidence from Spain, Italy, and Portugal. Infect Control Hosp Epidemiol. 2022 Oct;43(10):1360-1367. doi: 10.1017/ice.2021.429. Epub 2021 Oct 18. PMID: 34657648.
15. Singer M, Deutschman CS, Seymour CW, et al. The Third International Consensus Definitions for Sepsis and Septic Shock (Sepsis-3) *JAMA* 2016;315(8):801-10.
16. Calandra T, Cohen J.  International Sepsis Forum Definition of Infection in the ICU Consensus Conference. The international sepsis forum consensus conference on definitions of infection in the intensive care unit. *Crit Care Med* 2005;33(7):1538-48.
17. CDC. Antibiotic Resistance Threats in the United States, 2019. Atlanta, GA: U.S. Department of Health and Human Services, CDC; 2019. Available online at [www.cdc.gov/DrugResistance/Biggest-Threats.html](http://www.cdc.gov/DrugResistance/Biggest-Threats.html).
18. Strich JR, Heil EL, Masur H. Considerations for Empiric Antimicrobial Therapy in Sepsis and Septic Shock in an Era of Antimicrobial Resistance. J Infect Dis. 2020 Jul 21;222(Suppl 2):S119-S131. doi: 10.1093/infdis/jiaa221. PMID: 32691833; PMCID: PMC7372215.
19. Harbarth S, Nobre V, Pittet D. Does antibiotic selection impact patient outcome? Clin Infect Dis. 2007 Jan 1;44(1):87-93. doi: 10.1086/510075. Epub 2006 Nov 27. PMID: 17143822
20. Tamma PD, Hsu AJ. Defining the Role of Novel β-Lactam Agents That Target Carbapenem-Resistant Gram-Negative Organisms. J Pediatric Infect Dis Soc. 2019;8(3):251-260. doi:10.1093/jpids/piz002
21. WHO AWaRe (access, watch, reserve) classification of antibiotics for evaluation and monitoring of use, 2023. Available at: <https://www.who.int/publications/i/item/WHO-MHP-HPS-EML-2023.04>. Accessed March 19, 2024
22. European Centre for Disease Prevention and Control. Antimicrobial resistance in the EU/EEA (EARS-Net) - Annual Epidemiological Report 2022. Stockholm: ECDC; 2023.

**APPENDIX 1.**

**Investigator's statement.**

PREVALENCE STUDY ON ANTIMICROBIAL RESISTANCE: COLONIZATION AND INFECTION BY MULTIDRUG RESISTANT ORGANISMS AND ITS IMPACT ON MORTALITY IN ADULT AND PEDIATRIC CRITICAL CARE IN ARGENTINA. **(PREV-AR STUDY)**

Yours sincerely

I hereby declare that I undertake to carry out the study in (name of the institution)............ in accordance with the ethical principles governing research on human subjects, the considerations of the Declaration of Helsinki and the national regulations in force (Resolution 1480/11 of the National Ministry of Health and Provision 6677/10 of the National Administration of Medicines, Food and Medical Technology).

Sincerely yours,

Principal investigator's signature and date

**Letter of commitment signed by the hospital and critical area authorities to guarantee data collection throughout the study**.

I, (Name and surname)________ Medical Director by signing this document declare that I approve the inclusion of the Centre .................................... in the "STUDY OF **PREVALENCE** OF Colonization / INFECTION BY MULTI-RESISTANT MICROORGANISMS IN ADULT AND PEDIATRIC ICUs IN ARGENTINA **(PREV-AR) AND ITS IMPACT"** sponsored by the Argentine Society of Infectious Diseases and the Argentine Society of Intensive Care.

I, (Name and surname)________ Head of Critical Unit........... ......by signing this document I declare that I approve the inclusion of the requested data in the " PREVALENCE STUDY ON ANTIMICROBIAL RESISTANCE: COLONIZATION AND INFECTION BY MULTIDRUG RESISTANT ORGANISMS AND ITS IMPACT ON MORTALITY IN ADULT AND PEDIATRIC CRITICAL CARE IN ARGENTINA. **(PREV-AR STUDY)"** sponsored by the Argentine Society of Infectious Diseases and the Argentine Society of Intensive Care.

I will personally supervise this study, complying with the protocol that will be approved and providing complete and reliable information.

I agree to: 1. Execute this protocol in compliance with the institutional norms and current laws related to data protection of the participating subjects. Obtain ethical approval before making any changes to this project and report to the Committee any deviation from the protocol. 3. Inform the CIE in a timely manner of any unforeseen problem. 4. To communicate to the Committee the suspension of a study, sending a report with the results obtained and the reasons for suspension.

**APPENDIX 2. Case report forms**

**for hospitals and patients**

**PREVALENCE AND ASSOCIATED MORTALITY OF INFECTIONS BY MULTIDRUG-RESISTANT ORGANISMS IN ADULT INTENSIVE CARE UNITS IN ARGENTINA (PREV-AR**)

| **PARTICIPATING HOSPITAL INFORMATION**  **Name of the Institution:** | | | | | |
| --- | --- | --- | --- | --- | --- |
| **Province:** | | | **City:** | | |
| **Type of Hospital Public Private Community Health insurance Other** | | | | | |
| **Type of care provided by the hospital Polyvalent Univalent Pediatric Other** | | | | | |
| **Total number of beds available for utilization at the institution:** | | | | | |
| **Number of adult ICU beds participating in the study:** | | | | | |
| **Number of pediatric ICU beds participating in the study:** | | | | | |
| **PROGRAMMES** | | | | | |
| **Availability of infection prevention and control (IPC) committee** | **YES** (has an IPC committee that meets at least 3 times/year and carries out a IPC program) | | | | **No** (does not have the aforementioned resource) |
| **Availability of antimicrobial stewardship program** | **YES** (has a group dedicated to antimicrobial management that includes measurements of defined daily doses and/or days of treatment and actions that optimize the use of antimicrobials) | | | | **No** (does not have the aforementioned resource) |
| **SURVEILLANCE** | | | | | |
| **In your Institution, is active surveillance of MDRO colonization carried out?** | | | **No**  **YES** (fill in boxes in the next row) | | |
| **How frequently is surveillance carried out? (You can select more than one option)** | | **Weekly**  **Upon admission to the Institution**  **Pre-surgical** | **MDRO monitored (colonization) You can select more than one option** | ***S.aureus***  **Vancomycin-resistant *enterococci***  **Carbapenemase-**  **producing enterobacterales** | |
| **In your institution, is active surveillance of MDRO infections carried out?** | | | **No**  **YES** (fill in boxes in the next row) | | |
| **MDRO monitored for infection. You can select more than one option**  ***S.aureus* Vancomycin-resistant *enterococci***  **Carbapenemase- producing enterobacterales Extended-spectrum beta lactamases**  ***A.baumannii* resistant to carbapenems Difficult-to-treat *P. aeruginosa* *C. difficile*** | | | | | |
| **Methodology used to detect mechanisms of resistance (you can select more than one):** | | | **Phenotypic methods**  **Molecular methods**  **Immunochromatographic methods** | | |

PREVALENCE AND ASSOCIATED MORTALITY OF INFECTIONS BY MULTIDRUG-RESISTANT ORGANISMS IN ADULT INTENSIVE CARE UNITS IN ARGENTINA (PREV-AR)

| **CASE REPORT: All patients staying in the ICU on the day of the study must register on this form.**  **HOSPITAL CODE:_______________ PATIENT CODE: __________**  **PATIENT'S INITIALS:__ ADULT** □ **PEDIATRIC** □ |
| --- |
| **DATE OF BIRTH : __/__/__ SEX: M - F** |
| **Date of Hospital admission: __/__/__ of ICU admission: __/__/__ Date of ICU Discharge: __/__/__** |
| **OUTCOME**  **Deceased: YES □ NO** □ **Date of death: __/__/__** |
| **TYPE OF ADMISSION: MEDICAL** □ **Elective Surgical** □ **Emergency surgical** □  **TRAUMA: YES – NO INFECTION ON ADMISSION: YES – NO** |
| **RISK FACTORS FOR MDRO (IT CAN BE MORE THAN ONE):**   \| **Respiratory: Asthma/COPD** \| \| **YES** \| **NO** \| \| --- \| --- \| \| **Immunosuppressive treatment** \| \| **YES** \| **NO** \| \| --- \| --- \| \| \| --- \| --- \| --- \| --- \| --- \| --- \| --- \| --- \| \| **Diabetes** \| \| **YES** \| **NO** \| \| --- \| --- \| \| **Bone marrow transplant** \| \| **YES** \| **NO** \| \| --- \| --- \| \| \| **Obesity** \| \| **YES** \| **NO** \| \| --- \| --- \| \| **Solid organ transplantation** \| \| **YES** \| **NO** \| \| --- \| --- \| \| \| **Alcohol-related problem** \| \| **YES** \| **NO** \| \| --- \| --- \| \| **Hematology-oncology diseases** \| \| **YES** \| **NO** \| \| --- \| --- \| \| \| **Smoking** \| \| **YES** \| **NO** \| \| --- \| --- \| \| **Chemotherapy (last 6 months)** \| \| **YES** \| **NO** \| \| --- \| --- \| \| \| **Ischemic heart disease** \| \| **YES** \| **NO** \| \| --- \| --- \| \| **HIV** \| \| **YES** \| **NO** \| \| --- \| --- \| \| **YES** \| **NO** \| \| \| **Chronic liver disease** \| \| **YES** \| **NO** \| \| --- \| --- \| \| **Previous MDRO Colonization (last 6 months)** \| \| **YES** \| **NO** \| \| --- \| --- \| \| \| **Chronic renal failure** \| \| **YES** \| **NO** \| \| --- \| --- \| \| **Use of antibiotics (last 6 months)** \| \| **YES** \| **NO** \| \| --- \| --- \| \| |
| **SEVERITY OF DISEASE ON ICU ADMISSION:**  **APACHE II: ___ SOFA AT ICU ADMISSION: ___ SOFA at enrollment: ___** |
| **CLINICAL STATUS ON THE STUDY DAY (CHOOSE ONLY ONE CATEGORY):**   \| **1** \| **WITHOUT INFECTION** \| \| --- \| --- \| \| **2** \| **WIITH INFECTION BUT NO SEPSIS (NO ↑ of SOFA)** \| \| **3** \| **PROBABLE SEPSIS (↑SOFA ≥ 2 points, but with cultures that were finally negative.)** \| \| **4** \| **DEFINITE SEPSIS (↑SOFA ≥ 2 points with positive cultures)** \| \| **5** \| **SEPTIC SHOCK (requires vasopressors to maintain MAP≥65 mmHg after adequate resuscitation with fluids + lactate >2 mmol/L )** \| \| **6** \| **WITHOUT INFECTION, SEPSIS, OR SEPTIC SHOCK** \| |
| **Colonization**   \| **RECTAL SWAB** \| **Ca**rbapenemase-producing enterobacterales**:** not carried out □ positive □ negative □  Mechanisms of. resistance: KPC □ MBL □ KPC+MBL □ KPC+OXA □ MBL+OXA  Date of positive swab: ___/___/___ \| \| --- \| --- \| \| |
| **ORIGIN OF THE INFECTION: (IF PRESENT)**   \| **Community-acquired** \| \| **YES** \| **NO** \| \| --- \| --- \| \| **Hospital-acquired (non-ICU)** \| \| **YES** \| **NO** \| \| --- \| --- \| \| \| **SÍ** \| **NO** \| \| --- \| --- \| \| \| --- \| --- \| --- \| --- \| --- \| --- \| --- \| --- \| --- \| --- \| --- \| \| **ICU-acquired** \| \| **YES** \| **NO** \| \| --- \| --- \| \| **Long-term care** \| \| **YES** \| **NO** \| \| --- \| --- \| \| \| **SÍ** \| **NO** \| \| --- \| --- \| \| |
| **INFECTION N^0^ 1 DATE OF DIAGNOSIS: ___/__/__**   \| **Severe community-acquired pneumonia** \| \| **YES** \| **NO** \| \| --- \| --- \| \| **Community urinary tract infection** \| \| **YES** \| **NO** \| \| --- \| --- \| \| \| --- \| --- \| --- \| --- \| --- \| --- \| --- \| --- \| \| **Hospital-acquired pneumonia** \| \| **YES** \| **NO** \| \| --- \| --- \| \| **Urinary tract infection associated with a catheter** \| \| **YES** \| **NO** \| \| --- \| --- \| \| \| **Ventilator-associated pneumonia** \| \| **YES** \| **NO** \| \| --- \| --- \| \| **Nosocomial meningitis** \| \| **YES** \| **NO** \| \| --- \| --- \| \| \| **Bacteremia associated with a central venous catheter** \| \| **YES** \| **NO** \| \| --- \| --- \| \| **Surgical site infection** \| \| **YES** \| **NO** \| \| --- \| --- \| \| \| **Primary Bacteremia** \| \| **YES** \| **NO** \| \| --- \| --- \| \| **Intraabdominal infection** \| \| **YES** \| **NO** \| \| --- \| --- \| \| \| **Diarrhea from *C. difficile*** \| \| **YES** \| **NO** \| \| --- \| --- \| \| **Skin/soft tissue infection** \| \| **YES** \| **NO** \| \| --- \| --- \| \| \| **Cardiovascular infection** \| \| **YES** \| **NO** \| \| --- \| --- \| \| **Osteoarticular infection** \| \| **YES** \| **NO** \| \| --- \| --- \| \| \| **Obstetric or gynecological infection** \| \| **YES** \| **NO** \| \| --- \| --- \| \| **Other. Which one?** \| \| |
| **MICROORGANISM 1: Is it MDRO?** YES □ NO □.  **If it is MDRO, specify:** CRAB □ DT-PAE □ CPE □ ESBL □ VRE □ MRSA □  **Mechanisms of Resistance:** KPC □ MBL □ OXA □ KPC+MBL □KPC+OXA □ MBL+OXA BLEE □  **Identification of type of ESBL:** not carried out □ positive □ negative □ CTX M □ PER □ SHV □  **Detection of plasmid-mediated Amp C:** not carried out □ positive □ negative □  **IF NOT MDRO, tick the microorganism isolated**: *S. pneumoniae* □ *Pseudomonas* sp □ *S.aureus* □ *E.coli* □ Staphylococcus coagulase-negative □ *Proteus* sp.□ Klebsiella-Enterobacter-Serratia-Citrobacter □ Other: _____________ |
| **MICROORGANISM 2: Is it MDRO?** YES □ NO □.  **If it is MDRO, specify:** CRAB □ DT-PAE □ CPE □ ESBL □ VRE □ MRSA □  **Mechanisms of Resistance:** KPC □ MBL □ OXA □ KPC+MBL □KPC+OXA □ MBL+OXA BLEE □  **Identification of type of ESBL:** not carried out □ positive □ negative □ CTX M □ PER □ SHV □  **Detection of plasmid-mediated Amp C:** not carried out □ positive □ negative □  **IF NOT MDRO, tick the microorganism isolated**: *S. pneumoniae* □ *Pseudomonas* sp □ *S.aureus* □ *E.coli* □ Staphylococcus coagulase-negative □ *Proteus* sp.□ Klebsiella-Enterobacter-Serratia-Citrobacter □ Other: _____________ |
| **ANTIBIOTICS ADMINISTERED INFECTION N^0^ 1:**  1) Empiric □ Directed □  2) Adequate □ Inadequate □  3) Novel □ Traditional □  4) Antibiotics according to the WHO classification: Access □ Watch □ Reserve □ |
| **INFECTION N^0^ 2 DATE OF DIAGNOSIS: ___/__/__**   \| **Severe community-acquired pneumonia** \| \| **YES** \| **NO** \| \| --- \| --- \| \| **Community urinary tract infection** \| \| **YES** \| **NO** \| \| --- \| --- \| \| \| --- \| --- \| --- \| --- \| --- \| --- \| --- \| --- \| \| **Hospital-acquired pneumonia** \| \| **YES** \| **NO** \| \| --- \| --- \| \| **Urinary tract infection associated with a catheter** \| \| **YES** \| **NO** \| \| --- \| --- \| \| \| **Ventilator-associated pneumonia** \| \| **YES** \| **NO** \| \| --- \| --- \| \| **Nosocomial meningitis** \| \| **YES** \| **NO** \| \| --- \| --- \| \| \| **Bacteremia associated with a central venous catheter** \| \| **YES** \| **NO** \| \| --- \| --- \| \| **Surgical site infection** \| \| **YES** \| **NO** \| \| --- \| --- \| \| \| **Primary Bacteremia** \| \| **YES** \| **NO** \| \| --- \| --- \| \| **Intraabdominal infection** \| \| **YES** \| **NO** \| \| --- \| --- \| \| \| **Diarrhea from *C. difficile*** \| \| **YES** \| **NO** \| \| --- \| --- \| \| **Skin/soft tissue infection** \| \| **YES** \| **NO** \| \| --- \| --- \| \| \| **cardiovascular infection** \| \| **YES** \| **NO** \| \| --- \| --- \| \| **Osteoarticular infection** \| \| **YES** \| **NO** \| \| --- \| --- \| \| \| **Obstetric or gynecological infection** \| \| **YES** \| **NO** \| \| --- \| --- \| \| **Other. Which one?** \| \| |
| **MICROORGANISM 1: Is it MDRO?** YES □ NO □.  **If it is MDRO, specify:** CRAB □ DT-PAE □ CPE □ ESBL □ VRE □ MRSA □  **Mechanisms of Resistance:** KPC □ MBL □ OXA □ KPC+MBL □KPC+OXA □ MBL+OXA BLEE □  **Identification of type of ESBL:** not carried out □ positive □ negative □ CTX M □ PER □ SHV □  **Detection of plasmid-mediated Amp C:** not carried out □ positive □ negative □  **IF NOT MDRO, tick the microorganism isolated**: *S. pneumoniae* □ *Pseudomonas* sp □ *S.aureus* □ *E.coli* □ Staphylococcus coagulase-negative □ *Proteus* sp.□ Klebsiella-Enterobacter-Serratia-Citrobacter □ Other: _____________ |
| **MICROORGANISM 2: Is it MDRO?** YES □ NO □.  **If it is MDRO, specify:** CRAB □ DT-PAE □ CPE □ ESBL □ VRE □ MRSA □  **Mechanisms of Resistance:** KPC □ MBL □ OXA □ KPC+MBL □KPC+OXA □ MBL+OXA BLEE □  **Identification of type of ESBL:** not carried out □ positive □ negative □ CTX M □ PER □ SHV □  **Detection of plasmid-mediated Amp C:** not carried out □ positive □ negative □  **IF NOT MDRO, tick the microorganism isolated**: *S. pneumoniae* □ *Pseudomonas* sp □ *S.aureus* □ *E.coli* □ Staphylococcus coagulase-negative □ *Proteus* sp.□ Klebsiella-Enterobacter-Serratia-Citrobacter □ Other: _____________ |
| **ANTIBIOTICS ADMINISTERED INFECTION N^0^ 2:**  1) Empiric □ Directed □  2) Adequate □ Inadequate □  3) Novel □ Traditional □  4) Antibiotics according to the WHO classification: Access □ Watch □ Reserve □ |
| **INFECTION N^0^ 3 DATE OF DIAGNOSIS: ___/__/__**   \| **Severe community-acquired pneumonia** \| \| **YES** \| **NO** \| \| --- \| --- \| \| **Community urinary tract infection** \| \| **YES** \| **NO** \| \| --- \| --- \| \| \| --- \| --- \| --- \| --- \| --- \| --- \| --- \| --- \| \| **Hospital-acquired pneumonia** \| \| **YES** \| **NO** \| \| --- \| --- \| \| **Urinary tract infection associated with a catheter** \| \| **YES** \| **NO** \| \| --- \| --- \| \| \| **Ventilator-associated pneumonia** \| \| **YES** \| **NO** \| \| --- \| --- \| \| **Nosocomial meningitis** \| \| **YES** \| **NO** \| \| --- \| --- \| \| \| **Bacteremia associated with a central venous catheter** \| \| **YES** \| **NO** \| \| --- \| --- \| \| **Surgical site infection** \| \| **YES** \| **NO** \| \| --- \| --- \| \| \| **Primary Bacteremia** \| \| **YES** \| **NO** \| \| --- \| --- \| \| **Intraabdominal infection** \| \| **YES** \| **NO** \| \| --- \| --- \| \| \| **Diarrhea from *C. difficile*** \| \| **YES** \| **NO** \| \| --- \| --- \| \| **Skin/soft tissue infection** \| \| **YES** \| **NO** \| \| --- \| --- \| \| \| **cardiovascular infection** \| \| **YES** \| **NO** \| \| --- \| --- \| \| **Osteoarticular infection** \| \| **YES** \| **NO** \| \| --- \| --- \| \| \| **Obstetric or gynecological infection** \| \| **YES** \| **NO** \| \| --- \| --- \| \| **Other. Which one?** \| \| |
| **MICROORGANISM 1: Is it MDRO?** YES □ NO □.  **If it is MDRO, specify:** CRAB □ DT-PAE □ CPE □ ESBL □ VRE □ MRSA □  **Mechanisms of Resistance:** KPC □ MBL □ OXA □ KPC+MBL □KPC+OXA □ MBL+OXA BLEE □  **Identification of type of ESBL:** not carried out □ positive □ negative □ CTX M □ PER □ SHV □  **Detection of plasmid-mediated Amp C:** not carried out □ positive □ negative □  **IF NOT MDRO, tick the microorganism isolated**: *S. pneumoniae* □ *Pseudomonas* sp □ *S.aureus* □ *E.coli* □ Staphylococcus coagulase-negative □ *Proteus* sp.□ Klebsiella-Enterobacter-Serratia-Citrobacter □ Other: _____________ |
| **MICROORGANISM 2: Is it MDRO?** YES □ NO □.  **If it is MDRO, specify:** CRAB □ DT-PAE □ CPE □ ESBL □ VRE □ MRSA □  **Mechanisms of Resistance:** KPC □ MBL □ OXA □ KPC+MBL □KPC+OXA □ MBL+OXA BLEE □  **Identification of type of ESBL:** not carried out □ positive □ negative □ CTX M □ PER □ SHV □  **Detection of plasmid-mediated Amp C:** not carried out □ positive □ negative □  **IF NOT MDRO, tick the microorganism isolated**: *S. pneumoniae* □ *Pseudomonas* sp □ *S.aureus* □ *E.coli* □ Staphylococcus coagulase-negative □ *Proteus* sp.□ Klebsiella-Enterobacter-Serratia-Citrobacter □ Other: _____________ |
| **ANTIBIOTICS ADMINISTERED INFECTION N^0^ 3:**  1) Empiric □ Directed □  2) Adequate □ Inadequate □  3) Novel □ Traditional □  4) Antibiotics according to the WHO classification: Access □ Watch □ Reserve □ |

**APPENDIX 3**

The WHO list of Access, Watch, Reserve (AWaRe) classification of antibiotics for evaluation and monitoring of use, 2023

| **Antibiotic** | **Class** | **Category** |
| --- | --- | --- |
| Amikacin | Aminoglycosides | Access |
| Amoxicillin | Penicillins | Access |
| Amoxicillin/clavulanic-acid | Beta-lactam/beta-lactamase-inhibitor | Access |
| Ampicillin | Penicillins | Access |
| Ampicillin/sulbactam | Beta-lactam/beta-lactamase-inhibitor | Access |
| Arbekacin | Aminoglycosides | Watch |
| Aspoxicillin | Penicillins | Watch |
| Azidocillin | Penicillins | Access |
| Azithromycin | Macrolides | Watch |
| Azlocillin | Penicillins | Watch |
| Aztreonam | Monobactams | Reserve |
| Bacampicillin | Penicillins | Access |
| Bekanamycin | Aminoglycosides | Watch |
| Benzathine-benzylpenicillin | Penicillins | Access |
| Benzylpenicillin | Penicillins | Access |
| Biapenem | Carbapenems | Watch |
| Brodimoprim | Trimethoprim-derivatives | Access |
| Carbenicillin | Penicillins | Watch |
| Carindacillin | Penicillins | Watch |
| Carumonam | Monobactams | Reserve |
| Cefacetrile | First-generation-cephalosporins | Access |
| Cefaclor | Second-generation-cephalosporins | Watch |
| Cefadroxil | First-generation-cephalosporins | Access |
| Cefalexin | First-generation-cephalosporins | Access |
| Cefaloridine | First-generation-cephalosporins | Access |
| Cefalotin | First-generation-cephalosporins | Access |
| Cefamandole | Second-generation-cephalosporins | Watch |
| Cefapirin | First-generation-cephalosporins | Access |
| Cefatrizine | First-generation-cephalosporins | Access |
| Cefazedone | First-generation-cephalosporins | Access |
| Cefazolin | First-generation-cephalosporins | Access |
| Cefbuperazone | Second-generation-cephalosporins | Watch |
| Cefcapene-pivoxil | Third-generation-cephalosporins | Watch |
| Cefdinir | Third-generation-cephalosporins | Watch |
| Cefditoren-pivoxil | Third-generation-cephalosporins | Watch |
| Cefepime | Fourth-generation-cephalosporins | Watch |
| Cefetamet-pivoxil | Third-generation-cephalosporins | Watch |
| Cefiderocol | Other-cephalosporins | Reserve |
| Cefixime | Third-generation-cephalosporins | Watch |
| Cefmenoxime | Third-generation-cephalosporins | Watch |
| Cefmetazole | Second-generation-cephalosporins | Watch |
| Cefminox | Second-generation-cephalosporins | Watch |
| Cefodizime | Third-generation-cephalosporins | Watch |
| Cefonicid | Second-generation-cephalosporins | Watch |
| Cefoperazone | Third-generation-cephalosporins | Watch |
| Ceforanide | Second-generation-cephalosporins | Watch |
| Cefoselis | Fourth-generation-cephalosporins | Watch |
| Cefotaxime | Third-generation-cephalosporins | Watch |
| Cefotetan | Second-generation-cephalosporins | Watch |
| Cefotiam | Second-generation-cephalosporins | Watch |
| Cefoxitin | Second-generation-cephalosporins | Watch |
| Cefozopran | Fourth-generation-cephalosporins | Watch |
| Cefpiramide | Third-generation-cephalosporins | Watch |
| Cefpirome | Fourth-generation-cephalosporins | Watch |
| Cefpodoxime-proxetil | Third-generation-cephalosporins | Watch |
| Cefprozil | Second-generation-cephalosporins | Watch |
| Cefradine | First-generation-cephalosporins | Access |
| Cefroxadine | First-generation-cephalosporins | Access |
| Cefsulodin | Third-generation-cephalosporins | Watch |
| Ceftaroline-fosamil | Fifth-generation cephalosporins | Reserve |
| Ceftazidime | Third-generation-cephalosporins | Watch |
| Ceftazidime/avibactam | Third-generation-cephalosporins | Reserve |
| Cefteram-pivoxil | Third-generation-cephalosporins | Watch |
| Ceftezole | First-generation-cephalosporins | Access |
| Ceftibuten | Third-generation-cephalosporins | Watch |
| Ceftizoxime | Third-generation-cephalosporins | Watch |
| Ceftobiprole-medocaril | Fifth-generation cephalosporins | Reserve |
| Ceftolozane/tazobactam | Fifth-generation cephalosporins | Reserve |
| Ceftriaxone | Third-generation-cephalosporins | Watch |
| Cefuroxime | Second-generation-cephalosporins | Watch |
| Chloramphenicol | Amphenicols | Access |
| Chlortetracycline | Tetracyclines | Watch |
| Cinoxacin | Quinolones | Watch |
| Ciprofloxacin | Fluoroquinolones | Watch |
| Clarithromycin | Macrolides | Watch |
| Clindamycin | Lincosamides | Access |
| Clofoctol | Phenol derivatives | Watch |
| Clometocillin | Penicillins | Access |
| Clomocycline | Tetracyclines | Watch |
| Cloxacillin | Penicillins | Access |
| Colistin_IV | Polymyxins | Reserve |
| Colistin_oral | Polymyxins | Reserve |
| Dalbavancin | Glycopeptides | Reserve |
| Dalfopristin/quinupristin | Streptogramins | Reserve |
| Daptomycin | Lipopeptides | Reserve |
| Delafloxacin | Fluoroquinolones | Watch |
| Demeclocycline | Tetracyclines | Watch |
| Dibekacin | Aminoglycosides | Watch |
| Dicloxacillin | Penicillins | Access |
| Dirithromycin | Macrolides | Watch |
| Doripenem | Carbapenems | Watch |
| Doxycycline | Tetracyclines | Access |
| Enoxacin | Fluoroquinolones | Watch |
| Epicillin | Penicillins | Access |
| Eravacycline | Tetracyclines | Reserve |
| Ertapenem | Carbapenems | Watch |
| Erythromycin | Macrolides | Watch |
| Faropenem | Penems | Reserve |
| Fidaxomicin | Macrolides | Watch |
| Fleroxacin | Fluoroquinolones | Watch |
| Flomoxef | Second-generation-cephalosporins | Watch |
| Flucloxacillin | Penicillins | Access |
| Flumequine | Quinolones | Watch |
| Flurithromycin | Macrolides | Watch |
| Fosfomycin_IV | Phosphonics | Reserve |
| Fosfomycin_oral | Phosphonics | Watch |
| Furazidin | Nitrofuran derivatives | Access |
| Fusidic-acid | Steroid antibacterials | Watch |
| Garenoxacin | Fluoroquinolones | Watch |
| Gatifloxacin | Fluoroquinolones | Watch |
| Gemifloxacin | Fluoroquinolones | Watch |
| Gentamicin | Aminoglycosides | Access |
| Grepafloxacin | Fluoroquinolones | Watch |
| Hetacillin | Penicillins | Access |
| Iclaprim | Trimethoprim-derivatives | Reserve |
| Imipenem/cilastatin | Carbapenems | Watch |
| Imipenem/cilastatin/relebactam | Carbapenems | Reserve |
| Isepamicin | Aminoglycosides | Watch |
| Josamycin | Macrolides | Watch |
| Kanamycin_IV | Aminoglycosides | Watch |
| Kanamycin_oral | Aminoglycosides | Watch |
| Lascufloxacin | Fluoroquinolones | Watch |
| Latamoxef | Third-generation-cephalosporins | Watch |
| Lefamulin | Pleuromutilin | Reserve |
| Levofloxacin | Fluoroquinolones | Watch |
| Levonadifloxacin | Fluoroquinolones | Watch |
| Lincomycin | Lincosamides | Watch |
| Linezolid | Oxazolidinones | Reserve |
| Lomefloxacin | Fluoroquinolones | Watch |
| Loracarbef | Second-generation-cephalosporins | Watch |
| Lymecycline | Tetracyclines | Watch |
| Mecillinam | Penicillins | Access |
| Meropenem | Carbapenems | Watch |
| Meropenem/vaborbactam | Carbapenems | Reserve |
| Metacycline | Tetracyclines | Watch |
| Metampicillin | Penicillins | Access |
| Meticillin | Penicillins | Access |
| Metronidazole_IV | Imidazoles | Access |
| Metronidazole_oral | Imidazoles | Access |
| Mezlocillin | Penicillins | Watch |
| Micronomicin | Aminoglycosides | Watch |
| Midecamycin | Macrolides | Watch |
| Minocycline_IV | Tetracyclines | Reserve |
| Minocycline_oral | Tetracyclines | Watch |
| Miocamycin | Macrolides | Watch |
| Moxifloxacin | Fluoroquinolones | Watch |
| Nafcillin | Penicillins | Access |
| Nemonoxacin | Quinolones | Watch |
| Neomycin_IV | Aminoglycosides | Watch |
| Neomycin_oral | Aminoglycosides | Watch |
| Netilmicin | Aminoglycosides | Watch |
| Nifurtoinol | Nitrofuran derivatives | Access |
| Nitrofurantoin | Nitrofuran-derivatives | Access |
| Norfloxacin | Fluoroquinolones | Watch |
| Ofloxacin | Fluoroquinolones | Watch |
| Oleandomycin | Macrolides | Watch |
| Omadacycline | Tetracyclines | Reserve |
| Oritavancin | Glycopeptides | Reserve |
| Ornidazole_IV | Imidazoles | Access |
| Ornidazole_oral | Imidazoles | Access |
| Oxacillin | Penicillins | Access |
| Oxolinic-acid | Quinolones | Watch |
| Oxytetracycline | Tetracyclines | Watch |
| Panipenem | Carbapenems | Watch |
| Pazufloxacin | Fluoroquinolones | Watch |
| Pefloxacin | Fluoroquinolones | Watch |
| Penamecillin | Penicillins | Access |
| Penimepicycline | Tetracyclines | Watch |
| Pheneticillin | Penicillins | Watch |
| Phenoxymethylpenicillin | Penicillins | Access |
| Pipemidic-acid | Quinolones | Watch |
| Piperacillin | Penicillins | Watch |
| Piperacillin/tazobactam | Beta-lactam/beta-lactamase-inhibitor_anti-pseudomonal | Watch |
| Piromidic-acid | Quinolones | Watch |
| Pivampicillin | Penicillins | Access |
| Pivmecillinam | Penicillins | Access |
| Plazomicin | Aminoglycosides | Reserve |
| Polymyxin-B_IV | Polymyxins | Reserve |
| Polymyxin-B_oral | Polymyxins | Reserve |
| Pristinamycin | Streptogramins | Watch |
| Procaine-benzylpenicillin | Penicillins | Access |
| Propicillin | Penicillins | Access |
| Prulifloxacin | Fluoroquinolones | Watch |
| Ribostamycin | Aminoglycosides | Watch |
| Rifabutin | Rifamycins | Watch |
| Rifampicin | Rifamycins | Watch |
| Rifamycin_IV | Rifamycins | Watch |
| Rifamycin_oral | Rifamycins | Watch |
| Rifaximin | Rifamycins | Watch |
| Rokitamycin | Macrolides | Watch |
| Rolitetracycline | Tetracyclines | Watch |
| Rosoxacin | Quinolones | Watch |
| Roxithromycin | Macrolides | Watch |
| Rufloxacin | Fluoroquinolones | Watch |
| Sarecycline | Tetracyclines | Watch |
| Secnidazole | Imidazoles | Access |
| Sisomicin | Aminoglycosides | Watch |
| Sitafloxacin | Fluoroquinolones | Watch |
| Solithromycin | Macrolides | Watch |
| Sparfloxacin | Fluoroquinolones | Watch |
| Spectinomycin | Aminocyclitols | Access |
| Spiramycin | Macrolides | Watch |
| Streptoduocin | Aminoglycosides | Watch |
| Streptomycin_IV | Aminoglycosides | Watch |
| Streptomycin_oral | Aminoglycosides | Watch |
| Sulbactam | Beta-lactamase-inhibitors | Access |
| Sulbenicillin | Penicillins | Watch |
| Sulfadiazine | Sulfonamides | Access |
| Sulfadiazine/tetroxoprim | Sulfonamide-trimethoprim-combinations | Access |
| Sulfadiazine/trimethoprim | Sulfonamide-trimethoprim-combinations | Access |
| Sulfadimethoxine | Sulfonamides | Access |
| Sulfadimidine | Sulfonamides | Access |
| Sulfadimidine/trimethoprim | Sulfonamide-trimethoprim-combinations | Access |
| Sulfafurazole | Sulfonamides | Access |
| Sulfaisodimidine | Sulfonamides | Access |
| Sulfalene | Sulfonamides | Access |
| Sulfamazone | Sulfonamides | Access |
| Sulfamerazine | Sulfonamides | Access |
| Sulfamerazine/trimethoprim | Sulfonamide-trimethoprim-combinations | Access |
| Sulfamethizole | Sulfonamides | Access |
| Sulfamethoxazole | Sulfonamides | Access |
| Sulfamethoxazole/trimethoprim | Sulfonamide-trimethoprim-combinations | Access |
| Sulfamethoxypyridazine | Sulfonamides | Access |
| Sulfametomidine | Sulfonamides | Access |
| Sulfametoxydiazine | Sulfonamides | Access |
| Sulfametrole/trimethoprim | Sulfonamide-trimethoprim-combinations | Access |
| Sulfamoxole | Sulfonamides | Access |
| Sulfamoxole/trimethoprim | Sulfonamide-trimethoprim-combinations | Access |
| Sulfanilamide | Sulfonamides | Access |
| Sulfaperin | Sulfonamides | Access |
| Sulfaphenazole | Sulfonamides | Access |
| Sulfapyridine | Sulfonamides | Access |
| Sulfathiazole | Sulfonamides | Access |
| Sulfathiourea | Sulfonamides | Access |
| Sultamicillin | Beta-lactam/beta-lactamase-inhibitor | Access |
| Talampicillin | Penicillins | Access |
| Tazobactam | Beta-lactamase-inhibitors | Watch |
| Tebipenem | Carbapenems | Watch |
| Tedizolid | Oxazolidinones | Reserve |
| Teicoplanin | Glycopeptides | Watch |
| Telavancin | Glycopeptides | Reserve |
| Telithromycin | Macrolides | Watch |
| Temafloxacin | Fluoroquinolones | Watch |
| Temocillin | Penicillins | Watch |
| Tetracycline | Tetracyclines | Access |
| Thiamphenicol | Amphenicols | Access |
| Ticarcillin | Penicillins | Watch |
| Tigecycline | Glycylcyclines | Reserve |
| Tinidazole_IV | Imidazoles | Access |
| Tinidazole_oral | Imidazoles | Access |
| Tobramycin | Aminoglycosides | Watch |
| Tosufloxacin | Fluoroquinolones | Watch |
| Trimethoprim | Trimethoprim-derivatives | Access |
| Troleandomycin | Macrolides | Watch |
| Trovafloxacin | Fluoroquinolones | Watch |
| Vancomycin_IV | Glycopeptides | Watch |
| Vancomycin_oral | Glycopeptides | Watch |

**APPENDIX 4**

**Model letter for Hospital Ethics Committee**

City of ........, date

Dear members of the Ethics Committee of ..................,

I hereby submit for your consideration the PREVALENCE STUDY ON ANTIMICROBIAL RESISTANCE: Colonization AND INFECTION BY MULTIDRUG RESISTANT ORGANISMS AND ITS IMPACT ON MORTALITY IN ADULT AND PEDIATRIC CRITICAL CARE IN ARGENTINA. **(PREV-AR STUDY).**

It is a multicenter prevalence study with participation from several hospitals in different provinces of Argentina and is organized and sponsored by the Argentine Society of Infectious Diseases (SADI) and the Argentine Society of Intensive Care (SATI).

The main objective is to evaluate the number of patients colonized and infected by bacteria high-risk, multidrug-resistant bacteria and the impact on ICU mortality.

Thank you for your consideration and I am at your disposal for whatever you may require.

I would like to take this opportunity to send you my best regards.

Signature stamp.

**Model letter requesting an informed consent exception**

To whom it may concern:

This note is written with the purpose of expanding on the methodology outlined in the PREV-AR Study Protocol and provide justification for the exception of informed consent in this study.

The project has a descriptive design (there is no intervention of any kind) whose main objective is to evaluate the burden of infection and colonization by microorganisms in adult and pediatric critical care units and hospital mortality at 28 days.

The data collected will be analyzed and/or presented in a consolidated form and without identifying information.

Investigators will not enter any personally identifying information (PII) on enrolled patients (e.g., medical record number, surname and first name or national identification number) into the study database. Each patient will receive a study code and the association of study code and medical record number will be held by the principal investigator of the participating hospitals and utilized only for data corrections required by the study coordinators. Study coordinators will at not time have the patients' identifying data.

The non-PII data will be registered in the REDCAP platform, a database widely used in multicenter studies with proven reliability with respect to safeguarding the confidentiality of the information entered.

We hope that we have been able to justify the informed consent exception for the present study, we send you our best regards.

Principal investigators:

**Sample cover letter for hospital director**

City, of... of the month of the year:....

Mr. Director of institution X, Dr/a-------------

I hereby inform you that I have submitted for consideration to the Hospital's Ethics Committee the PREVALENCE STUDY ON ANTIMICROBIAL RESISTANCE: Colonization AND INFECTION BY MULTIDRUG RESISTANT ORGANISMS AND ITS IMPACT ON MORTALITY IN ADULT AND PEDIATRIC CRITICAL CARE IN ARGENTINA. **(PREV-AR STUDY)**.

It is a multicenter prevalence study with participation from several hospitals in different provinces of Argentina and is organized and sponsored by the Argentine Society of Infectious Diseases (SADI) and the Argentine Society of Intensive Care (SATI).

The main objective is to evaluate the number of patients colonized and infected by bacteria considered to be high-risk and multidrug-resistant and the impact on mortality.

I would like to take this opportunity to send my best regards and am at your disposal for any further information you may require.

Signature stamp.
